# *BNC2* is a putative tumor suppressor gene in high-grade serous ovarian carcinoma and impacts cell survival after oxidative stress

**DOI:** 10.1038/cddis.2016.278

**Published:** 2016-09-22

**Authors:** Laura Cesaratto, Eleonora Grisard, Michela Coan, Luigi Zandonà, Elena De Mattia, Elena Poletto, Erika Cecchin, Fabio Puglisi, Vincenzo Canzonieri, Maria Teresa Mucignat, Antonella Zucchetto, Gabriele Stocco, Alfonso Colombatti, Milena S Nicoloso, Riccardo Spizzo

**Affiliations:** 1 Division of Experimental Oncology2, Department of Translational Research, Centro di Riferimento Oncologico (CRO Aviano), National Cancer Institute, Aviano, Italy; 2 Department of Life and Reproduction Sciences, University of Verona, Verona, Italy; 3 Division of Experimental and Clinical Pharmacology, Department of Translational Research Centro di Riferimento Oncologico (CRO Aviano), National Cancer Institute, Aviano, Italy; 4 Department of Oncology, University Hospital of Udine, Udine, Italy; 5 Department of Medical and Biological Sciences, University of Udine, Udine, Italy; 6 Division of Pathology, Department of Translational Research, CRO Aviano National Cancer Institute, Aviano, Italy; 7 Clinical and Experimental Onco-Hematology Unit, Centro di Riferimento Oncologico (CRO Aviano), National Cancer Institute, Aviano, Italy; 8 Department of Life Sciences, University of Trieste, Trieste, Italy

## Abstract

Rs3814113 is the single-nucleotide polymorphism (SNP) showing the strongest association with high-grade serous ovarian carcinoma (HGSOC) incidence and is located in an intergenic region about 44 kb downstream of basonuclin 2 (*BNC2*) gene. Lifetime number of ovulations is associated with increased risk to develop HGSOC, probably because of cell damage of extrauterine Müllerian epithelium by ovulation-induced oxidative stress. However, the impact of low-penetrance HGSOC risk alleles (e.g. rs3814113) on the damage induced by oxidative stress remains unclear. Therefore, the purpose of this study was to investigate whether rs3814113 genetic interval regulates *BNC2* expression and whether *BNC2* expression levels impact on cell survival after oxidative stress. To do this, we analyzed gene expression levels of *BNC2* first in HGSOC data sets and then in an isogenic cell line that we engineered to carry a 5 kb deletion around rs3814113. Finally, we silenced BNC2 and measured surviving cells after hydrogen peroxide (H_2_O_2_) treatment to simulate oxidative stress after ovulation. In this paper, we describe that *BNC2* expression levels are reduced in HGSOC samples compared with control samples, and that BNC2 expression levels decrease following oxidative stress and ovulation *in vitro* and *in vivo*, respectively. Moreover, deletion of 5 kb surrounding rs3814113 decreases *BNC2* expression levels in an isogenic cell line, and silencing of *BNC2* expression levels increases cell survival after H_2_O_2_ treatment. Altogether, our findings suggest that the intergenic region located around rs3814113 regulates *BNC2* expression, which in turn affects cell survival after oxidative stress response. Indeed, HGSOC samples present lower *BNC2* expression levels that probably, in the initial phases of oncogenic transformation, conferred resistance to oxidative stress and ultimately reduced the clearance of cells with oxidative-induced damages.

Epithelial ovarian cancer (EOC) is a relatively uncommon tumor accounting for ~2.6% of newly diagnosed tumors in 2015 in the United States; however, EOC is the fifth most common cause of cancer death in women in the United States and in the Western countries, and the first among gynecological tumors.^1^ High-grade serous ovarian carcinoma (HGSOC) is the single largest group of EOC and the histotype that accounts for almost two-thirds of ovarian cancer deaths. HGSOC is typically diagnosed in postmenopausal age and at advanced clinical stages (III and IV), mainly owing to the lack of early symptoms and of affordable screening programs applicable to the general population.^2^ For these reasons, it is urgent to discover the key molecular events that predispose to and/or occur in the early phases of HGSOC oncogenic transformation, which are necessary for the development of effective screening tools, chemoprevention programs^3, 4^ and eventually novel anticancer therapies.^5^

Recent reports elucidated novel details about the origin of HGSOC: studies from women carrying *BRCA1/2* mutations identified tubal intraepithelial carcinoma in the distal fimbriated end of the fallopian tube as the probable precursor lesion of HGSOC.^6^ This may also occur in the secondary Müllerian system locations (e.g. ovary and peritoneum) that, along with fallopian tube, belong to the extrauterine Müllerian epithelium.^7, 8^

In HGSOC, the most important risk factor is the occurrence of the disease in a first-degree relative;^9^ this excess of risk is because of both environmental (e.g. lifetime number of ovulations, body mass index and so on) and genetic factors. Concerning environmental factors, lifetime number of ovulations was one of the first to be described,^10^ and rodent models of repeated superovulation confirmed the insurgence of modifications resembling early transformation.^11, 12^ The explanation of the association between lifetime number of ovulations and HGSOC incidence may reside in cell damage by ovulation-induced oxidative stress.^11, 13^ Concerning genetic risk factors, which have the greatest impact, less than half of the excess of risk is due to *BRCA1/2* high penetrance mutations, whereas the remaining risk is probably because of low- to moderate-penetrance risk alleles.^14^ Low- to moderate-penetrance risk alleles (e.g. single-nucleotide polymorphism (SNP)) can be identified by genome-wide association studies (GWAS)^15^ and ultimately give novel molecular insights on tumor biology.^16, 17, 18^

SNP rs3814113 has the strongest association with HGSOC incidence^14^ and is located in an intergenic region of human 9p22.2 locus, ~44 kb downstream from the transcription starting point (TSS) of basonuclin 2 (*BNC2*) gene and ~200 kb upstream from TSS of centlein (*CNTLN*) gene. *BNC2* transcribes a zinc-finger protein highly conserved during evolution,^19^ with about 200 transcript isoforms,^20^ and *BNC2-*knockout (KO) mice die within 24 h after birth.^21^ Preliminary evidences pointed to *BNC2* as the coding transcript that could be responsible for the association between 9p22.2 locus and HGSOC: *BNC2* promoter is more frequently methylated in peripheral lymphomonocytes of EOC patients compared with healthy subjects,^22^ *BNC2* expression levels are reduced in EOC cell lines compared with primary human ovarian surface epithelial cell cultures,^23^ and *BNC2* was described as a presumptive tumor suppressor gene in bladder, esophageal and glioblastoma tumors.^24, 25, 26^

The impact of low-penetrance HGSOC risk alleles (e.g. rs3814113) on cell damage induced by oxidative stress remains unclear. For instance, acquired genetic modifications, which reduce cell death after oxidative stress, may offer fallopian serous epithelial cells the advantage to survive despite carrying genetic damages caused by oxidative stress exposure.^12^ Therefore, the purpose of this study was to investigate whether rs3814113 genetic interval regulates *BNC2* expression and whether *BNC2* expression levels impact cell survival after oxidative stress. To do this, first we engineered an isogenic cell line to carry a 5 kb deletion around rs3814113 and measured *BNC2* levels; next, we silenced *BNC2* expression levels, and after hydrogen peroxide (H_2_O_2_) treatment, which simulates oxidative stress after ovulation,^11^ we measured cell survival.

## Results

### *BNC2* is in linkage with rs3814113 and is a putative tumor suppressor in HGSOC

**Figure 1:**
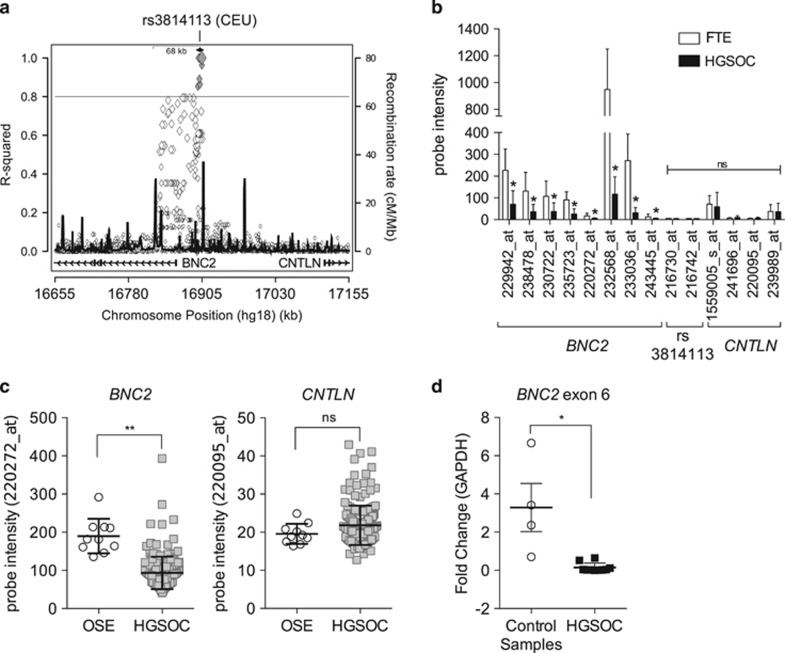
Rs3814113 at chromosome 9p22.2 is in linkage with BNC2, a putative tumor suppressor gene in HGSOC. (**a**) Regional LD plot generated using the SNP Annotation and Proxy Search web interface using rs3814113 as target SNP (https://www.broadinstitute.org/mpg/snap/ldsearch.php). The left-hand y axis shows values for *r*^2^ (correlation coefficient, a measure of LD; 0.8 was set as threshold), whereas the right-hand y axis shows recombination rate values in centiMorgans (units of recombination) per million bases (cM/Mb). Diamond labels correspond to SNP (black diamond is rs3814113, gray filled diamonds represent SNPs with *r*^2^≥ 0.8 with rs3814113, empty filled diamonds have an *r*^2^<0.8). Higher peaks of recombination rate indicate an association between rs314113 and the end of *BNC2* genetic locus. (**b**) Analyses of gene expression for Affymetrix probes surrounding rs3814113 in the 9p22.2 locus from the GSE10971 data set. Affymetrix probes are indicated by a number followed by ‘_at'. Laser capture microdissected non-malignant distal FTE *n*=24; HGSOC, *n*=13. *BNC2*, intergenic region surrounding rs3814113, and *CNTLN* loci were represented in this array by 8, 2 and 4 probes, respectively. Bars are mean±S.E. (**c**) Analyses of *BNC2* and *CNTLN* expression in the GSE26712 data set. Non-malignant ovarian surface epithelium (OSE), *n*=10; HGSOC, *n*=185. In this Affymetrix array, *BNC2* and *CNTLN* were represented only by one probe (220272_at and 22095_at, respectively). Lines are mean±S.E. (**d**) qRT-PCR analysis of *BNC2* and *CNTLN* gene expression levels normalized by *GAPDH* in an original cohort of samples. Control samples=non-malignant ovarian and fallopian specimens, *n*=4; HGSOC samples, *n*=10. Scatter dot plot bars indicate mean±S.E. **P<0.01; *P<0.05; ns, nonsignificant. *t*-Test assuming unequal variances was used for statistical analysis

To consolidate that *BNC2* is the principal actor of the association between human 9p22.2 locus and HGSOC incidence, we used two independent strategies. First, we searched for SNPs in linkage disequilibrium (LD) with rs3814113 by using SNAP bioinformatics tool,^27^ and we discovered that rs3814113 is correlated (*r*^2^≥0.8) to other 11 SNPs located within 6.8 kb upstream (Figure 1a and Supplementary Table 1). Yet, recombination rates suggested a larger haplotypic block, which includes the first exon and intron of *BNC2*, embracing one of *BNC2* promoters, and includes additional SNPs that correlated to rs3814113 with an *r*^2^≥0.5 (Figure 1a and Supplementary Table 1). Within the identified haplotypic block, rs3814113 was the only SNP present in the Illumina SNP-chromatin immunoprecipitation (ChIP) array used in the GWAS study that initially correlated 9p22.2 region with EOC predisposition.^14^

In the second strategy, we searched for genes within the 9p22.2 locus differentially expressed between tumor-free control and HGSOC samples. To do this, we interrogated the gene data set by Tone *et al.*,^28^ which analyzed microdissected non-malignant fallopian tube epithelium (FTE) and HGSOC samples using a high-density Affymetrix gene expression array (Affymetrix Human Genome U133 Plus 2.0 Array, Santa Clara, CA, USA). We correlated signal intensity levels of microarray probes located within ±4 Mb from rs3814113 (Supplementary Figure S1A) with the sample type (i.e. FTE or HGSOC samples), and we found that *BNC2* locus was the region with the greatest number of probes inversely correlated with HGSOC (i.e. lower signal intensity in HGSOC compared with non-malignant FTE samples) (Supplementary Figure S1B and Figure 1b). Instead, *CNTLN*, which is the closest gene downstream of rs3814113, was not differentially expressed between the groups (Figure 1b). We confirmed these results in a second publically available data set^29^ (Figure 1c). Meanwhile, we collected at our Institute an original cohort of HGSOC and tumor-free control samples (fallopian tube and ovarian), in which we confirmed by quantitative real-time PCR (qRT-PCR) that *BNC2* expression was reduced in HGSOC (Figure 1d). Therefore, both LD and gene expression data analyses pointed to *BNC2* as the most likely candidate genetic element involved in HGSOC predisposition.

**Figure 2:**
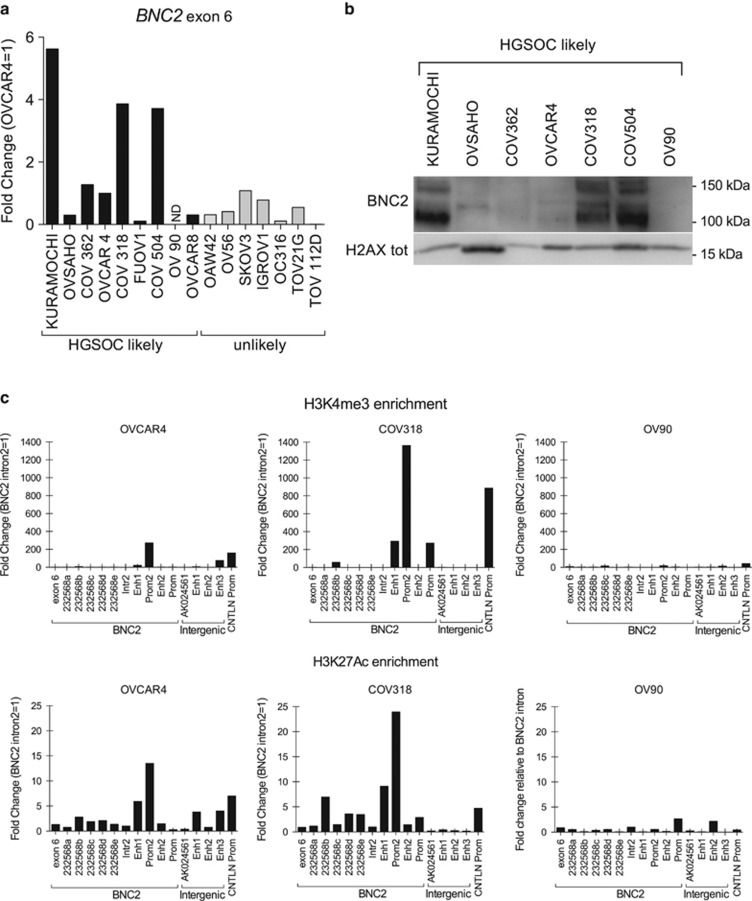
BNC2 expression and genetic regulation. (**a**) qRT-PCR analysis of *BNC2* expression in a panel of 16 ovarian cancer cell lines, grouped in likely and unlikely HGSOC. (**b**) Western blot detection of BNC2 shows several isoforms in NP40-insoluble protein extracts from seven likely HGSOC cell lines. H2AX total was used as loading control. (**c**) ChIP for histone mark enrichment (measured by qRT-PCR) in the putative promoter/enhancer genomic regions comprising *BNC2*, *CNTLN* and the intergenic region in between, according to UCSC (Supplementary Figure S3). H3K4me3 (top panels) and H3K27Ac (bottom panels)=histone H3 trymethylated on lysine 4 and acetylated on lysine 27, respectively. BNC2 intron 2 (Intr 2) was set as 1

Next, we measured *BNC2* transcript expression in a panel of 16 EOC cell lines (Figure 2a). By grouping EOC cells into likely and unlikely HGSOC,^30^ we did not observe any significant difference in *BNC2* expression levels between the two groups. In seven likely HGSOC cell lines, BNC2 protein expression was proportional to mRNA expression and showed several isoforms between 100 and 150 kDa (Figure 2b); moreover, BNC2 was localized exclusively in the NP40 nuclear insoluble fraction (Supplementary Figures S2A and B) in agreement with Vanhoutteghem and Djian.^19^ Interestingly, of the 12 EOC cell lines that we genotyped for rs3814113, all carried the T allele in homozygosis (data not shown), which is the allele associated with increased EOC predisposition in the general population,^14^ whereas according to dbSNP build 141,^31^ the expected frequency for rs3814113 in the general population is 58% for T and 42% for C.

According to Vanhoutteghem and Djian^20^ and Ghanbari *et al.*,^32^ *BNC2* has several putative promoters and enhancers, which is also confirmed by the Encode project^33^ (Supplementary Figure S3); however, histone marks enriched in promoters and enhancers (H3K4me3 and H3K27Ac, respectively) have never been described in HGSOC cell models. Therefore, we performed ChIP for these histone marks in OV90, OVCAR4 and COV318 EOC cell lines. Consistent with *BNC2* mRNA and protein expression levels (Figures 2a and b), histone mark enrichment in candidate genetic regions was almost absent in OV90, intermediate in OVCAR4 and high in COV318 (Figure 2c). Moreover, in both COV318 and OVCAR4, the region labeled as BNC2 Prom2 had the highest enrichment, in agreement with Ghanbari *et al.*^32^

To sum up, these data indicate that the genomic region in LD (r^2^≥0.5) with rs3814113 includes one of the *BNC2* promoters and that *BNC2* is downregulated in HGSOC samples compared with non-malignant FTE samples.

### A human genomic region including rs3814113 regulates BNC2 expression

**Figure 3:**
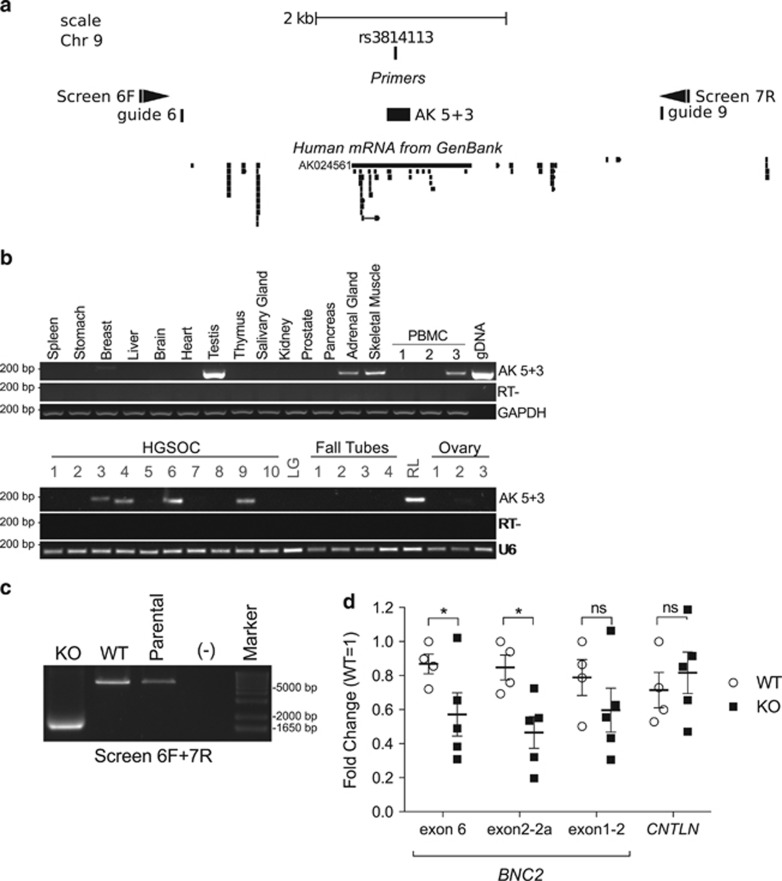
A human genomic region including rs3814113 regulates BNC2 expression. (**a**) Genomic view of the 6.6 kb, surrounding rs3814113, with annotated human mRNAs from GenBank, according to UCSC genome browser. Positions for guides 6 and 9 (guide RNAs used for CRISPR (clustered regularly interspaced short palindromic repeat) deletion of the region), for Screen 6F and Screen 7F (primers used for PCR screening of KO), and for AK 5+3 (amplicon used to detect *AK024561* transcript) are indicated. (**b**) Agarose gel of PCR with AK 5+3 primers to detect *AK024561* in human samples: top panels, 14 normal human tissues and 3 PBMCs from healthy donors; bottom panels, 11 serous ovarian cancers (10 high grade, 1 low grade=LG) and 8 non-malignant control samples (4 fallopian tubes, 1 round ligament=RL, 3 ovaries). gDNA=genomic DNA used as PCR positive control; RT-PCR on non-retrotranscribed RNA, to exclude genomic contamination; *GAPDH* and *U6*=housekeeping genes used for normalization. (**c**) Representative agarose gel of PCR with Screen 6F+Screen 7R primer pair showing the deletion of the 5 kb region surrounding rs3814113. (**d**) qRT-PCR analysis of *BNC2* and *CNTLN* expression in 293FT KO cell clones. Points for WT and KO samples represent normalized expression data from four distinct WT and five distinct KO clones. The values for each clone are the average of two biologically independent experiments. Three distinct primer pairs (encompassing BNC2's exon 1-2, exon 2-2a and exon 6) were used to detect *BNC2* expression levels. *t*-Test assuming unequal variances was used for statistical analysis. Scatter dot plot bars indicate mean±S.E. *P<0.05. ns, nonsignificant

Interestingly, rs3814113 resides inside *AK024561* genomic locus, a putative non-coding RNA transcript that was cloned in human adipose tissue according to GenBank records (Figure 3a). Two probes of the Affymetrix U133 Plus 2.0 Array hybridize to *AK024561* (i.e. 216730_at and 216742_at) (Supplementary Figure 3), but both show low expression signals and no difference between non-malignant FTE and HGSOC samples in Tone *et al.*^28^ (Figure 1b and Supplementary Figure S1B). We evaluated expression levels of *AK024561* in a panel of 17 different non-malignant tissues, including peripheral blood mononuclear cells (PBMCs) collected from healthy donors, and in a panel of HGSOC samples, fallopian tube and ovary tumor-free samples. *AK024561* was strongly expressed only in the testis^20^ and moderately expressed in the adrenal gland and skeletal muscle (Figure 3b). Only in 4 out of 10 HGSOC tumor samples, we could detect a faint band, whereas we did not detect any band in the fallopian tube or ovarian healthy samples, except in one sample obtained from the round ligament (Figure 3b). We evaluated *AK024561* expression in several cell lines of mesodermal origin, the same origin of the extrauterine Müllerian epithelium (COV318, COV504, COV362, OVCAR4, 293FT, JURKAT, MAVER, MEC, EHEB, HL60, RAJI, K562, SKUT-1, U2OS, HT1080, human mesenchymal stem cells, MET5A), but none showed any detectable expression levels except COV318, which had an extremely low signal (data not shown). Consistently, we did not observe histone mark enrichment in the intergenic regions including *AK024561* locus and nearby enhancers (intergenic enhancers 1, 2 and 3), except for a H3K27Ac enrichment in OVCAR4 intergenic enhancers 1 and 3 (Figure 2c).

To investigate whether *AK024561* locus may still regulate *BNC2* expression, we deleted 5 kb surrounding the SNP (between guide 6 and guide 9 in Figure 3a) using CRISPR-Cas9 system.^17, 34^ Unfortunately, we could not obtain KO cell clones in COV318, which is the only cell line, among the ones that we tested, that expressed *AK024561* (data not shown), because of the low efficiency of COV318 to grow as single-cell clones; therefore, we opted for 293FT because of very high efficiency of transfection and of single-cell clone recovery. We recovered five clones with homozygous deletion of the targeted region (KO clones) (Figure 3c and Supplementary Figure S4B), and we compared them with four wild-type (WT) clones, which had either point mutations in guide RNA target sites (*n*=2) (guides 6 and 9 in Figure 3a) or no mutations (*n*=2) (sequences provided in the Supplementary Material). We measured *BNC2* and *CNTLN* transcript expression levels in WT and KO clones; given the multiplicity of *BNC2* isoforms,^20^ we designed three qRT-PCR primer pairs that target exon boundary 1-2, 2-2a and exon 6^20^ (Supplementary Figure S3). By these means, we observed that the deletion of the 5 kb region comprising rs3814113 almost halved expression levels of *BNC2* but not of *CNTLN* (Figure 3d). Unfortunately, 293FT expression levels of BNC2 protein were almost undetectable and it was not possible to observe any difference between WT and KO clones. Of note, in 293FT cells, rs3814113 is heterozygous (C/T) and *AK024561* was never detected both in whole and nuclear RNA (data not shown).

Taken together, these findings indicate that the genetic region encompassing rs3814113 regulates *BNC2* but not *CNTLN* expression levels, and that this regulation does not depend on *AK024561* transcription.

### Oxidative stress reduces BNC2 expression *in vitro* and *in vivo*

**Figure 4:**
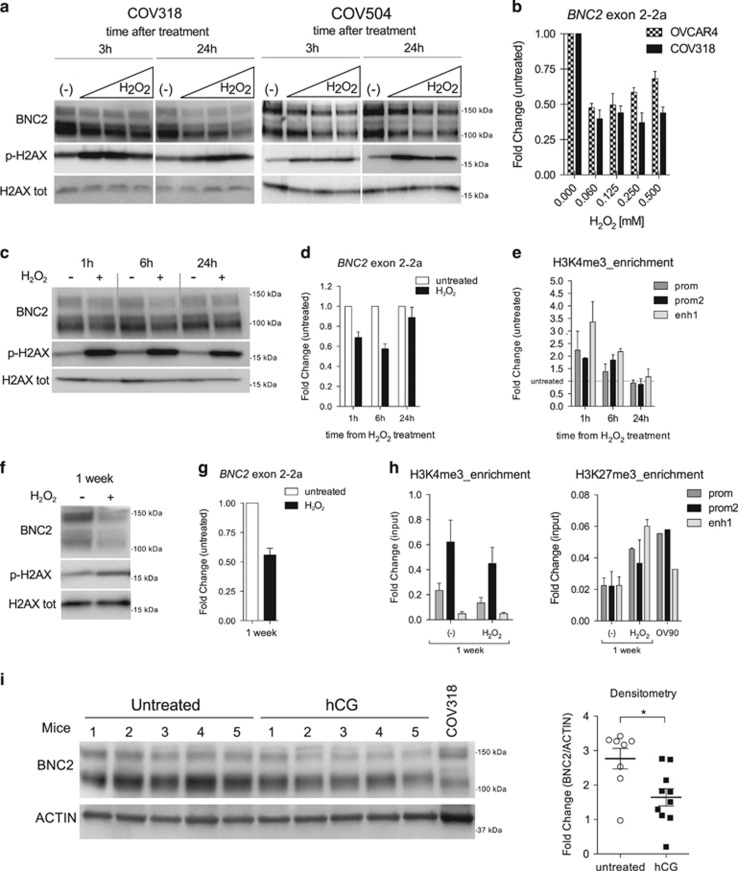
Oxidative stress reduces BNC2 expression *in vitro* and *in vivo*. (**a**) Western blot detection of BNC2 protein levels in NP40-insoluble extracts from COV318 and COV504 cells treated with increasing amounts of H_2_O_2_ (0.06, 0.125 and 0.25 mM) at 3 or 24 h post-treatment. Total H2AX was used as loading control. Phospho-H2AX (pH2AX) was detected to check effectiveness of H_2_O_2_ treatment. (**b**) qRT-PCR analysis of *BNC2* expression in COV318 and OVCAR4 at 5 h after H_2_O_2_ treatment. Bars indicate mean±S.E. from two independent experiments. (**c**) Western blot detection of BNC2 protein levels in NP40-insoluble extracts from COV318 cells treated with 0.06 mM H_2_O_2_ and collected 1, 6 and 24 h post-treatment. Total H2AX was used as loading control. pH2AX was detected to check effectiveness of H_2_O_2_ treatment. (**d**) qRT-PCR analysis of *BNC2* expression in COV318 at 1, 6 and 24 h after 0.06 mM H_2_O_2_ treatment. Bars indicate mean±S.E. from two independent experiments. (**e**) ChIP for H3K4me3 enrichment (measured by qRT-PCR) of three regulatory elements of *BNC2* locus in COV318 treated with 0.06 mM H_2_O_2_ at different time points. Bars indicate mean±S.E. from two independent experiments. (**f**) Western blot detection of BNC2 protein levels in NP40-insoluble extracts from COV318 cells treated with 0.06 mM H_2_O_2_ and collected 1 week post-treatment. Total H2AX was used as loading control. pH2AX was detected to check effectiveness of H_2_O_2_ treatment. (**g**) qRT-PCR analysis of *BNC2* expression in COV318 at 1 week after 0.06 mM H_2_O_2_ treatment. Bars indicate mean±S.E. from two independent experiments. (**h**) ChIP for H3K4me3 enrichment (left panel) and H3K27me3 enrichment (right panel) (measured by qRT-PCR) of three regulatory elements of *BNC2* locus in COV318 at 1 week after 0.06 mM H_2_O_2_ treatment. Bars indicate mean±S.E. from two independent experiments. (**i**) Left panel: Western blot detection of BNC2 protein levels in NP40-insoluble extracts from prepubertal mice oviducts treated or not with hCG 16 h earlier. COV318 was used as positive control for BNC2 expression. Right panel: Densitometric analysis of BNC2 western blot bands in oviducts from hCG treated (*n*=10) and untreated (*n*=10) prepubertal mice as described above. Scatter dot plot bars indicate mean±S.E. from two independent experiments, including the one presented in the left panel. *P<0.05

The incessant ovulation model of EOC origin lies on the hypothesis that fallopian tubes and ovaries are exposed to increased levels of reactive oxygen species (ROS) during ovulation cycle, which reiterated over time may generate the soil for EOC insurgence.^4, 10, 12^ Therefore, to investigate whether BNC2 may be involved in the incessant ovulation model, we evaluated BNC2 expression levels following oxidative stress. To simulate postovulation environment, we treated OV90, COV318 and COV504 with increasing doses of H_2_O_2_ and evaluated BNC2 levels at early and late time points, after 3 h and 24 h, respectively.^35^ Effectiveness of H_2_O_2_ cell treatment was verified by phosphorylation of H2AX with no evident disparities among cell lines (Figure 4a and Supplementary Figure 5). While in OV90 BNC2 levels remained undetectable even after H_2_O_2_ treatment (data not shown), in COV318 and COV504, we observed a dosage-dependent reduction of BNC2 protein levels at 24 h after treatment, but not at earlier time points. To understand whether BNC2 reduction occurred both at the transcriptional and protein level, we measured *BNC2* by qRT-PCR and found that, 5 h following H_2_O_2_ exposure, *BNC2* transcript levels were already halved, suggesting a regulation of *BNC2* at the transcriptional/mRNA level rather than at the protein stability level (Figure 4b). To address deeper the molecular mechanism by which *BNC2* is regulated in an oxidative stress condition, we investigated the role of epigenetic modifications in *BNC2* expression at different time points after H_2_O_2_ exposure. To do this, we performed a time-course experiment in COV318 treated with 0.06 mM of H_2_O_2_, which is the lowest dosage at which we previously observed a reduction of BNC2 protein expression levels, and we confirmed that BNC2 expression decreases both at mRNA and protein levels (Figures 4c and d). As *BNC2* promoter contains three CpG islands (Supplementary Figure S3) and *BNC2* promoter methylation has been previously found associated with EOC,^22^ we analyzed methylation status of CpG island 141 (chr9:16870124–16872020 hg19) and CpG island 21 (chr9:16828936–16829169 hg19) and did not observe any methylation both in untreated and treated cells (data not shown). Concomitantly, we evaluated activating and repressing histone modifications (H3K4me3 and H3K27me3, respectively) in the same time-course experiment. Unexpectedly, activating H3K4me3 modification increased at earlier time points and eventually decreased at 24 h (Figure 4e), whereas we did not find any enrichment of repressing H3K27me3 modification (data not shown). We also wondered whether *BNC2* repression persisted longer than 24 h after H_2_O_2_ exposure; indeed, we found *BNC2* downregulation both at mRNA and protein levels lasting up to 1 week post-treatment (Figures 4f and g), likely due to a decrease of activating H3K4me3 enrichment and an increase of suppressing H3K27me3 in BNC2 promoters and enhancers (Figure 4h). Enrichment of H3K27me3 modification in H_2_O_2_- treated COV318 cells was similar to OV90, which constitutively do not express BNC2 (Figure 4h). According to our results, decrease of *BNC2* expression after H_2_O_2_ exposure is not due to methylation and histone modifications at early time points, whereas at later time points, histone modification is involved in *BNC2* transcription repression.

To study the effect of ovulation on BNC2 expression levels *in vivo*, we induced superovulation in prepubertal mice and measured BNC2 protein levels in murine oviducts. In this model, BNC2 displayed several protein isoforms similarly to COV318, and its protein expression levels decreased in ovulated mice compared with control mice (Figure 4i).

To sum up, these evidences suggest that BNC2 expression levels decrease both *in vitro* and *in vivo* after oxidative stress or ovulation, respectively.

### *BNC2* silencing increases ovarian cell survival following H_2_O_2_ treatment

**Figure 5:**
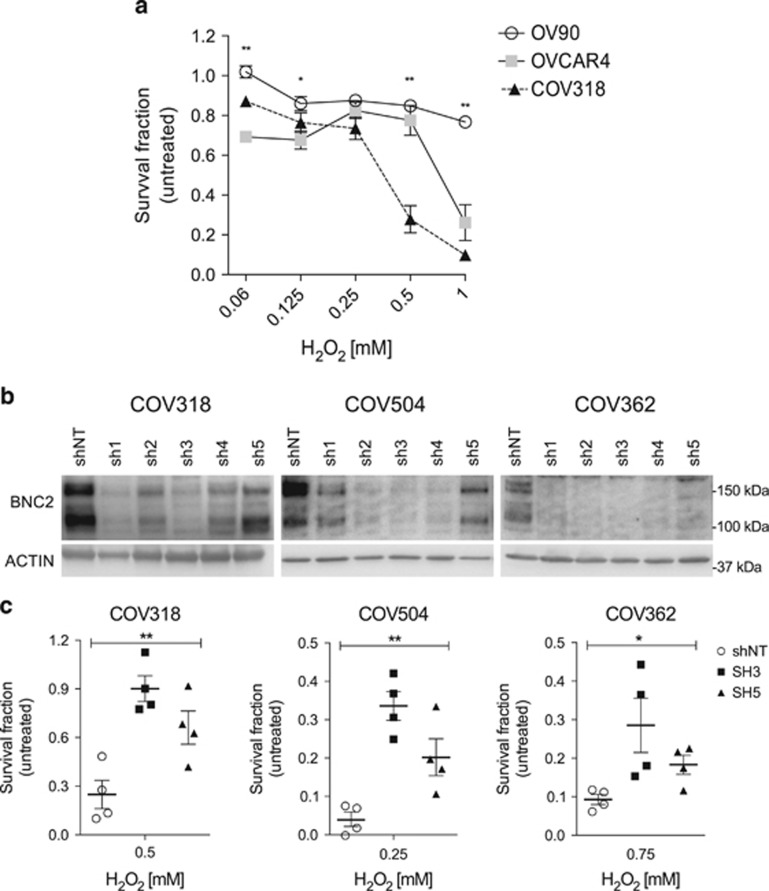
*BNC2* silencing increases ovarian cancer cell survival following H_2_O_2_ treatment. (**a**) MTT viability, expressed as fold change compared with untreated cells (=1), of OV90, OVCAR4 and COV318 cells 24 h following H_2_O_2_ treatment. **P<0.01, *P<0.05 using *t*-test assuming unequal variances. Points in graph represent average±S.E. of three independent biological replicates. (**b**) Western blot detection of BNC2 protein levels in NP40-insoluble extracts from COV318, COV362 and COV504 expressing five different short hairpins (sh) targeting *BNC2*. shNT=sh non-target, negative control. (**c**) Scatter dot plot resume MTT viability experiments, expressed as fold change compared with untreated cells (=1), of COV318, COV362 and COV504 silenced (sh3 and sh5) or not (shNT) for BNC2 24 h following H_2_O_2_ treatment. **P<0.01, *P<0.05 using one-way analysis of variance (ANOVA) test. Scatter dot plot bars indicate mean±S.E. of four replicates (two independent biological experiments performed in duplicate). Results from different H_2_O_2_ treatments for each cell line are shown: COV318 (0.5 mM), COV362 (0.75 mM) and COV504 (0.25 mM)

Next, we argued whether BNC2 levels affect cell survival after oxidative stress. To this aim, we treated OV90 (no BNC2 expression), OVCAR4 (intermediate BNC2 expression) and COV318 (high BNC2 expression) (Figure 2b) with increasing doses of H_2_O_2_; 24 h after treatment, we measured surviving cells by MTT assay. Interestingly, we observed that OV90 cells are resistant to H_2_O_2_ treatment (>80% alive); at the same dosages, OVCAR4 and COV318 showed a degree of H_2_O_2_ sensitivity that was inversely correlated with BNC2 expression levels (Figure 5a). With this observation in mind, we silenced BNC2 levels by shRNA in three different BNC2-expressing HGSOC cell lines and in BNC2-null OV90 cell line. We tested five short hairpins spanning different regions of *BNC2* (Supplementary Figure S3), and used the ones with a strong and intermediate silencing ability (sh3 and sh5) for further experiments (Figure 5b and Supplementary Figure S6A). When we treated BNC2-silenced cells with H_2_O_2_, we observed that silenced cells were more resistant to H_2_O_2_ compared with control cells in three HGSOC cell lines (Figure 5c); this effect was not recapitulated in OV90 (Supplementary Figure 6B), which are null for BNC2, indicating that the phenotype that we described was BNC2-specific. Cells silenced with sh5, which always displayed the smallest BNC2 reduction, were also the ones with the smallest increase in cell survival, implying that the effect that we observed was dependent on BNC2 expression levels.

In conclusion, these evidences indicate that low BNC2 expression levels have a protective role in response to H_2_O_2_, which is in line with reduced expression levels of BNC2 in HGSOC compared with control samples (Figures 1b–d).

## Discussion

Our study showed that the first intron and exon of *BNC2* and one of its promoters are in linkage with rs3814113, and that *BNC2* expression levels were reduced in HGSOC samples compared with control samples. Moreover, we observed that deletion of 5 kb surrounding rs3814113 decreased *BNC2* expression levels in an isogenic cell line and, finally, that silencing of BNC2 expression levels increased HGSOC cell survival after H_2_O_2_ treatment.

Altogether, our findings give new insights about the mechanism at the basis of HGSOC and 9p22.2 association,^14^ and in particular they suggest that the intergenic region located around rs3814113 regulates *BNC2* expression, which in turn affects cell survival after oxidative stress response. Indeed, HGSOC samples present lower *BNC2* expression levels that, in the initial phases of oncogenic transformation, may confer resistance to oxidative stress and ultimately reduce the clearance of cells with ROS-induced damages.

So far, few groups have published results regarding the role of *BNC2* in cancer, but all suggested a putative tumor suppressor function of *BNC2*,^24, 25, 26^ in agreement with our findings. Ramus *et al.*^36^ reported that rs3814113 minor allele (C) is also associated to a lower risk of EOC in *BRCA1*/2-mutated populations. When we analyzed the gene expression data set from Tone *et al.*,^28^ which contains both *BRCA1/2* WT and mutant control samples, we did not observe any difference in BNC2 expression levels between the two groups (data not shown). Ultimately, these evidences may suggest that *BNC2* locus and *BRCA1/2* loci are two independent risk factors in EOC onset.

Our study presents some limitations: for instance, (1) we used cell lines derived from HGSOC and not cell lines derived from normal tissues (e.g. normal fallopian tubes); nevertheless, we confirmed our *in vitro* findings in the normal oviducts obtained from a mouse model of superovulation. (2) We did not investigate in our cell model the allele-specific impact of rs3814113 on *BNC2* expression, as we expected that it would have taken a GWAS-size clones to observe any effect. Instead, we took advantage of an isogenic cell line knocked out for 5 kb interval surrounding rs3814113 to obtain a stronger effect. Despite having found a link between rs3814113 genomic region and *BNC2* expression, we cannot exclude that other genetic elements that are located in the haplotypic block of rs3814113 participate in *BNC2* regulation. (3) Finally, even though we described that silencing of *BNC2* expression affects survival after H_2_O_2_ treatment, despite our attempts, we could not generate cells overexpressing BNC2 to rescue silencing experiments and we did not dissect in greater detail the molecular mechanism at the basis of this phenotype. Precisely, our results on BNC2 localization in agreement with^19^ suggest that BNC2 is localized in the nucleus more firmly compared with transcription factors and is a putative chromatin-modulating protein; therefore, ChIP-seq would be a good approach to study BNC2 binding regions.

EOC and breast cancer share some common risk factors (e.g. *BRCA1/2* status); therefore, it might be expected that BNC2 has a role also in breast cancer onset. We are not aware of BNC2 expression levels in normal and tumor breast samples; however, there is no report of any association between rs3814113 and breast cancer risk, in both the general population^15^ and *BRCA1/2* mutant carriers,^36^ ultimately suggesting that *BNC2* is not involved in breast cancer.

According to our results, *BNC2* seems to be a good candidate to develop new molecular tools to select healthy subjects with increased risk of EOC. Despite having demonstrated that the 5 kb region around rs3814113 regulates *BNC2* expression, it is unlikely that we can use rs3814113 genotyping as an indicator of *BNC2* expression^14^ and novel strategies should be undertaken. At the same time, we showed that BNC2 silencing affects cell survival after oxidative stress, which suggests that modulating BNC2 levels pharmacologically may be an intriguing strategy to reduce the excess of EOC risk in selected populations (e.g. *BRCA1*/2 mutation carriers). Ultimately, if we consider that the discovery of high penetrance risk alleles (*BRCA1/2* mutations) not only helped to identify subjects at higher risk but it was also helpful to select EOC patients benefiting the most from poly(ADP-ribose) polymerase inhibitors,^5^ the same scenario may also be true for BNC2. Hence, understanding how to target BNC2 and/or the associated stress response pathway could lead to the development of alternative therapeutic approaches for patients with HGSOC.

## Materials and Methods

### Cell lines, cell cultures, lentiviral transduction and transfections

Cell lines used in this work are listed in Supplementary Table 2, which includes cell type, source and culture media. No antibiotics were routinely used for cell culture. Cells have been authenticated in 2012 by BMR Genomics (Padova, Italy) according to Cell ID System (Promega, Madison, WI, USA) and using Genemapper ID version 3.2.1, to identify DNA STR profiles. Cells were expanded and have been used only for a short number of passages after authentication. Cell lines were maintained at 37 °C under 5% CO_2_ in humidified incubators and routinely tested using MycoAlert Detection Kit (Lonza, Cologne, Germany) for mycoplasma contamination. Only mycoplasma-negative cells were used for experiments.

BNC2-silenced ovarian cancer cell lines were generated by lentiviral transduction. Briefly, 293FT cells were co-transfected with pLKO.1 puro vectors encoding for either human *BNC2*-shRNAs (sh1_TRCN0000108177, sh2_TRCN0000327841 sh3_TRCN0000108179, sh4_TRCN0000108175, sh5_TRCN0000108176, MISSION shRNAs from Sigma-Aldrich (St. Louis, MO, USA)) or non-target shRNA control (SHC016; Sigma-Aldrich), together with ViraPower Packaging Mix (pLP1, pLP2 and pLP/VSV-G) (Invitrogen, Thermo Fisher Scientific, Eugene, OR, USA). Viral supernatants were collected 72 h later and transducing units per ml were determined by limiting dilution titration in HCT116 cells. An MOI of ~5 was used for transducing ovarian cancer cells. After 5 days of selection with puromycin, BNC2-silenced cells were immediately used for *in vitro* experiments, protein and RNA extraction. Polybrene (Sigma-Aldrich) at a final concentration of 8 *μ*g/ml was used to increase transduction efficiency.

Cell transfections were performed using Lipofectamine 2000 reagent (Invitrogen, Thermo Fisher Scientific) according to the manufacturer's instructions.

### CRISPR-Cas9 system

KO of *AK024561* in 293FT cells was generated by CRISPR-Cas9 system, according to Ran *et al.*,^34^ using two guide RNAs (guides 6 and 9; Supplementary Table 3) that delimit a region of 5 kb surrounding the *AK024561* non-coding transcript (Figure 3a). To this end, guide RNAs were cloned into pSpCas9(BB)-2A-GFP (PX458) vector (cat. no. 48138; Addgene, Cambridge, MA, USA) and transfected into cells. To evaluate guide RNA-directed Cas9 cutting efficiency, genomic DNA was extracted using Gentra Puregene Cell Kit (Qiagen Sciences, Germantown, MD, USA), according to the manufacturer's instructions, and was subjected to Surveyor Assay using Surveyor Mutation Detection Kit (IDT Integrated DNA Technologies, Leuven, Belgium), as per the manufacturer's instructions (Supplementary Figure 4A).

The 293FT CRISPR-modified cell clones were grown following GFP single-cell sorting by FACS, then genomic DNA was extracted and clones were screened by PCR, using two primers, AK024561 5+3 and Screen 6F+7R (Figure 3a and Supplementary Table 3), which for KO clones were expected to yield no product and a 1.6 kb fragment, respectively (Figure 3c and Supplementary Figure S4). Genetic deletion was confirmed by direct Sanger sequencing, using BigDye Terminator v.3.1 Cycle Sequencing Kit (Applied Biosystems, Foster City, CA, USA). Sequences of all the clones that we used are reported in Supplementary Material.

### Cell treatments

H_2_O_2_ treatment was performed as follows: cells plated 24 h earlier in a 6- or 12-well format were exposed for 1 h to H_2_O_2_ (0–4 mM range) in serum-free medium. After two washes with PBS, H_2_O_2_ containing medium was removed and replaced with complete medium. Cell viability was assed 24 h later by MTT staining (M2003; Sigma-Aldrich). MTT stock solution was added to cells at a final concentration of 0.28 mg/ml and incubated for 1 h at 37 °C. Supernatant was then discarded and cells were air-dried. Reduced MTT, measure of cellular metabolic activity and index of cell viability was dissolved by adding 1 or 2 ml of DMSO, depending on plate format. Two hundred microliters of DMSO were transferred to a 96-well and absorbance was measured at 570 nm with Infinite M1000 Microplate Reader (Tecan, Männedorf, Switzerland).

### Western blotting

To obtain protein extracts, cells were incubated for 20 min on ice in lysis buffer (0.5% NP40, 50 mM HEPES, 250 mM NaCl, 5 mM EDTA, 0.5 mM EGTA, 1 *μ*M DTT), containing Protease Inhibitors (Complete; Roche, Mannheim, Germany) and Phosphatase Inhibitor Cocktail 1 and 2 (P2850 and P5726; Sigma-Aldrich), vortexing every 5 min. Cell lysates were clarified by centrifugation at a maximum speed for 10 min (NP40-soluble fraction). Cell pellet was then suspended in SDS lysis buffer (4% SDS, 100 mM Tris-HCl (pH 6.8), 20% glycerol) and boiled at 95 °C for 10 min (NP40-insoluble fraction, containing chromatin-bound proteins, such as BNC2). Protein concentration of the NP40-soluble fraction was quantified using the Protein Assay Dye Reagent Concentrate (Bio-Rad, Hercules, CA, USA), whereas the concentration of the NP40-insoluble fraction was empirically derived from its corresponding soluble fraction.

For immunoblotting analysis, ~40 *μ*g of proteins were separated using 4–20% SDS-PAGE Criterion TGX Stain-Free Precast Gels (Bio-Rad) and transferred to PVDF membranes (Bio-Rad). Membranes were blocked with 5% non-fat dried milk in TBS-0.1% Tween-20, and incubated overnight with the following antibodies: BNC2 (HPA018525; Sigma-Aldrich), ACTIN (cat. no. A2066; Sigma-Aldrich), histone H2AX (cat. no. 2595; Cell Signaling, Danvers, MA, USA) and phospho-histone H2AX (Ser139) (20E3) (cat. no. 2577; Cell Signaling). Incubation with ECL rabbit IgG, HRP-linked whole antibody (GE Heathcare Lifesciences, Little Chalfont, UK) was performed at room temperature for 1 h at a dilution of 1 : 3000. According to signal intensity, either ECL Western Blotting Detection Reagents RPN 2106 (GE Healthcare) or Luminata Forte Western HRP Substrate (Merck Millipore, Darmstadt, Germany) were used for secondary antibody detection using Chemidoc MP Imager (Bio-Rad). Bands were analyzed and quantified using Image Lab v.5.2 (Bio-Rad).

### ChIP assay

Before harvesting, 3x10^7^ cells were crosslinked with 1% (v/v) of formaldehyde in complete culture medium for 10 min at room temperature and then quenched with 125 mM glycine. Cell pellets were washed two times with ice-cold PBS and then lysed in 1 ml of ChIP lysis buffer (1% SDS, 50 mM Tris-HCl (pH 8.1), 10 mM EDTA), completed with Protease Inhibitors (Complete; Roche) and Phosphatase Inhibitor Cocktail 1 (P5726; Sigma-Aldrich). Lysates were sonicated with an ultrasonic homogenizer Sonopuls 3200 (Bandelin, Berlin, Germany) for 12 cycles (30 s ON+60 s OFF) and clarified by centrifugation. DNA fragment size was checked in a 2% agarose gel with a 100 bp DNA marker. Fifty micrograms of protein extract, diluted 10-fold in ChIP dilution buffer (0.01% SDS, 1.1% Triton X-100, 1.2 mM EDTA, 16.7 mM Tris-HCl (pH 8.1) and 167 mM NaCl), were incubated, for 2 h with rotating at 4 °C, with 2 *μ*g of the following specific ChIP grade antibodies: anti-histone H3-acetyl K27, H3 tri-methyl K4 (cat. no. ab4729, ab8580; Abcam, Cambridge, UK) and H3 tri-methyl K27) (cat. no. 9733; Cell Signaling). Immunoprecipitation was performed by using Protein A Sepharose 4 Fast Flow (GE Healthcare) overnight at 4 °C. After several washes of the IP samples (in low salt, in high salt and in LiCl wash buffers), the elution was performed incubating in 1% SDS and 100 mM NaHCO_3_ for 45 min with rotation at room temperature. To reverse crosslinking, supernatants were collected and incubated at 65 °C overnight with the addition of NaCl, 200 mM final concentration. Samples were subjected to DNA extraction using Gentra Puregene Cell Kit (Qiagen) according to the manufacturer's instructions. DNA levels for the 9p22.2 regions of interest were quantified by qRT-PCR using specific primers (Supplementary Table 3).

### RNA extraction, cDNA synthesis and qRT-PCR

RNA was extracted using the Isol-RNA lysis reagent (5 Prime, Hamburg, Germany) according to protocol instructions. Extraction was followed by PCA (phenol: chloroform: isoamyl alcohol 25  :  24 : 1; Sigma-Aldrich) purification that reduces salt contamination and by DNase digestion (Turbo-Dnase, Ambion, Thermo Fisher Scientific). RNA quality was assessed using agarose gel electrophoresis after RNA exposure to 70 °C for 5 min. cDNA was synthesized from 1 *μ*g of RNA using the AMV Reverse Transcriptase (Promega) with random primers (Promega). cDNA was diluted 10 times and used for qRT-PCR using iQ SYBR Green Supermix (Bio-Rad) with the appropriate primers (Supplementary Table 3). qRT-PCR reactions were carried out either in a 96-well optical reaction plates using Two-Color Real-Time PCR Detection System MyiQ2 (Biorad) or in 384-well optical reaction plates using Applied Biosystems 7900HT Fast Real-Time PCR System (Thermo Fisher Scientific), according to manufacturer's protocol. The 2^−ΔΔCt^ method was used to calculate the relative abundance of RNA genes, measuring *GAPDH* expression as housekeeping control.

Primers used in this work were designed using Primer 3 Plus,^37^ purchased from Sigma-Aldrich and are listed in Supplementary Table 3.

### *In vivo* experiments

Prepubertal FVB female mice (between 3 and 5 weeks of age) were stimulated with 5 *μ*IU of serum gonadotropin (Folligon, Schering-Plough Animal Health, Upper Hutt, Wellington, New Zealand) and 48 h later with 5 *μ*IU of human chorionic gonadotropin (Chorulon, MSD Animal Health, Madison, NJ, USA) or with placebo (0.9% NaCl) by intraperitoneal injection. After 16 h, mice were humanely culled, assessed for ovulation and oviducts were collected. NP40-soluble and -insoluble proteins were extracted from oviducts, as described above. Animal experiments were reviewed and approved by the CRO Aviano's Institutional Animal Care and Use Committee and were conducted according to committee's guidelines.

### Patient samples

Samples were obtained at CRO Aviano, National Cancer Institute (Aviano, Italy) according to Cervo *et al.*,^38^ after informed consent forms were signed, through CRO-Biobank facility. Scientific use of biological material was approved by CRO Aviano's Ethics Committee and Internal Review Board. Control ovarian and fallopian tube samples were collected from women undergoing annexectomy for diseases other than ovarian and fallopian tubes cancers, whereas HGSOC specimens were collected from ovarian cancer patients undergoing first-line cytoreductive surgery (by laparoscopy or laparotomy). CRO Aviano's Pathology Unit performed sample pathologic evaluation as part of standard routine on fresh-frozen specimens and confirmed a tumor percentage >70%.

### Statistical and gene expression analysis

Graphs presented in figures were obtained using the GraphPad Prism v.6.0d software (La Jolla, CA, USA) and statistical analysis were carried out using the JMP v.9.0.1 software (Carry, NC, USA). Data were examined using the two-tailed Student's *t*-test assuming unequal variances or one-way ANOVA test when we compared more than two groups at once. Differences were considered significant at *P*<0.05 and *P*<0.01 and labeled accordingly (* or **, respectively).

Data sets used for gene expression analysis include Gene Expression Omnibus IDs: GSE10971^28^ and GSE26712.^29^ For each set, gene signal values were background corrected, normalized by the quantile method and reported as log 2-transformed values, as implemented in the rma function of the Affy R package.

## Abbreviations

HGSOC: high-grade serous ovarian carcinoma
SNP: single-nucleotide polymorphism
BNC2: basonuclin 2
H_2_O_2_: hydrogen peroxide
EOC: epithelial ovarian cancer
TIC: tubal intraepithelial carcinoma
BRCA1/2: breast cancer
GWAS: genome-wide association studies
TSS: transcription starting point
CNTLN: centlein gene
LD: linkage disequilibrium
FTE: fallopian tube epithelium
H3K4me3: histone 3 lysine 4 tri-methylation
H3K27Ac: histone 3 lysine 27 acetylation
ChIP: chromatin immunoprecipitation
PBMC: peripheral blood mononuclear cell
CRISPR: clustered regularly interspaced short palindromic repeat
ROS: reactive oxygen species
