# Supplementary material for: BNC2 is a putative tumor suppressor gene in high-grade serous ovarian carcinoma and impacts cell survival after oxidative stress
Source: Cell Death Dis. 2016 Sep 22;7(9):e2374–. doi: 10.1038/cddis.2016.278 (PMC5059877; doi:10.1038/cddis.2016.278)
Supplement: Supplementary Information [file cddis2016278x1.doc]

# Title

***BNC2* is a putative tumor suppressor gene in high-grade serous ovarian carcinoma and impacts cell survival after oxidative stress.**

**Running Title**

**Bnc2 impacts oxidative stress outcome in EOC cells**

**Authors**

Laura Cesaratto^1*^, Eleonora Grisard^1*^, Michela Coan^1,8^, Luigi Zandonà^1^, Elena De Mattia^2^, Elena Poletto^3^, Erika Cecchin^2^, Fabio Puglisi^3-4^, Vincenzo Canzonieri^5^, Maria Teresa Mucignat^1^, Antonella Zucchetto^6^, Gabriele Stocco^7^, Alfonso Colombatti^1^, Milena S. Nicoloso^1^ and Riccardo Spizzo^1^.

**Authors’ affiliation:**

^1^ Division of Experimental Oncology 2, Department of Translational Research, Centro di Riferimento Oncologico (CRO Aviano), National Cancer Institute, Aviano, PN, Italy

^2^ Division of Experimental and Clinical Pharmacology, Department of Translational Research Centro di Riferimento Oncologico (CRO Aviano), National Cancer Institute, Aviano, PN, Italy

^3^ Department of Oncology, University Hospital of Udine, Udine, Italy

^4^ Department of Medical and Biological Sciences, University of Udine, Udine, Italy

^5^ Division of Pathology, Department of Translational Research, CRO Aviano National Cancer Institute, Aviano, PN, Italy

^6^ Clinical and Experimental Onco-Hematology Unit, Centro di Riferimento Oncologico (CRO Aviano), National Cancer Institute, Aviano, PN, Italy

^7^ Department of Life Sciences, University of Trieste, Trieste, Italy

^8^ Department of Life and Reproduction Sciences, University of Verona, Verona, Italy

* These authors equally contributed to this original article.

Correspondence: Riccardo Spizzo (rspizzo@cro.it) and Milena Sabrina Nicoloso (mnicoloso@cro.it)

**Authors’ email:**

Laura Cesaratto [lacesaratto@gmail.com](mailto:lacesaratto@gmail.com)

Luigi Zandonà [luzando@libero.it](mailto:luzando@libero.it)

Michela Coan [michela.coan@gmail.com](mailto:michela.coan@gmail.com)

Eleonora Grisard [eleonoragrisard@gmail.com](mailto:eleonoragrisard@gmail.com)

Elena De Mattia [edemattia@cro.it](mailto:edemattia@cro.it)

Elena Poletto [polettoelena@libero.it](mailto:polettoelena@libero.it)

Cecchin Erika [ececchin@cro.it](mailto:ececchin@cro.it)

Fabio Puglisi [fabio.puglisi@uniud.it](mailto:fabio.puglisi@uniud.it)

Vincenzo Canzonieri [vcanzonieri@cro.it](mailto:vcanzonieri@cro.it)

Maria Teresa Mucignat mtmucignat@cro.it

Antonella Zucchetto zucchetto.soecs@cro.it

Gabriele Stocco [stoccog@units.it](mailto:stoccog@units.it)

Colombatti Alfonso [acolombatti@cro.it](mailto:acolombatti@cro.it)

Milena S. Nicoloso [mnicoloso@cro.it](mailto:mnicoloso@cro.it)

Riccardo Spizzo [rspizzo@cro.it](mailto:rspizzo@cro.it)

**Supplementary Information include**

Supplementary Table 1

Supplementary Table 2

Supplementary Table 3

Supplementary Figure S1

Supplementary Figure S2

Supplementary Figure S3

Supplementary Figure S4

Supplementary Figure S5

Supplementary References

Sanger Sequences of CRISPR clones

# Supplementary Table 1

SNP correlation with rs3814113 according to SNAP server.

| **TARGET SNP** | **Proxy** | **Distance** | **RSquared** | **Chromosome** | **Coordinate_HG18** |
| --- | --- | --- | --- | --- | --- |
| **rs3814113** | **rs3814113** | **0** | **1** | **chr9** | **16905021** |
| **rs3814113** | **rs7032221** | **126** | **1** | **chr9** | **16904895** |
| **rs3814113** | **rs6475092** | **1548** | **1** | **chr9** | **16903473** |
| **rs3814113** | **rs4445329** | **3264** | **1** | **chr9** | **16901757** |
| **rs3814113** | **rs4366169** | **3383** | **1** | **chr9** | **16901638** |
| **rs3814113** | **rs7045767** | **4123** | **1** | **chr9** | **16900898** |
| **rs3814113** | **rs10738468** | **4258** | **1** | **chr9** | **16900763** |
| **rs3814113** | **rs10962684** | **5688** | **1** | **chr9** | **16899333** |
| **rs3814113** | **rs10810671** | **186** | **0.963** | **chr9** | **16904835** |
| **rs3814113** | **rs10738467** | **4344** | **0.892** | **chr9** | **16900677** |
| **rs3814113** | **rs10465044** | **3356** | **0.864** | **chr9** | **16901665** |
| **rs3814113** | **rs55689948** | **6852** | **0.853** | **chr9** | **16898169** |
| rs3814113 | rs36116821 | 14256 | 0.794 | chr9 | 16890765 |
| rs3814113 | rs10962668 | 20881 | 0.794 | chr9 | 16884140 |
| rs3814113 | rs3927680 | 27655 | 0.794 | chr9 | 16877366 |
| rs3814113 | rs10810657 | 30435 | 0.794 | chr9 | 16874586 |
| rs3814113 | rs12345776 | 33144 | 0.794 | chr9 | 16871877 |
| rs3814113 | rs10962643 | 57618 | 0.781 | chr9 | 16847403 |
| rs3814113 | rs28498684 | 14326 | 0.764 | chr9 | 16890695 |
| rs3814113 | rs10810650 | 41470 | 0.764 | chr9 | 16863551 |
| rs3814113 | rs2153271 | 50500 | 0.736 | chr9 | 16854521 |
| rs3814113 | rs1339552 | 66231 | 0.736 | chr9 | 16838790 |
| rs3814113 | rs74664507 | 1185 | 0.724 | chr9 | 16903836 |
| rs3814113 | rs10962679 | 9580 | 0.718 | chr9 | 16895441 |
| rs3814113 | rs10756819 | 56937 | 0.717 | chr9 | 16848084 |
| rs3814113 | rs1416742 | 58138 | 0.71 | chr9 | 16846883 |
| rs3814113 | rs12350739 | 30004 | 0.709 | chr9 | 16875017 |
| rs3814113 | rs10962672 | 16902 | 0.697 | chr9 | 16888119 |
| rs3814113 | rs10122763 | 36528 | 0.682 | chr9 | 16868493 |
| rs3814113 | rs7029285 | 11073 | 0.68 | chr9 | 16893948 |
| rs3814113 | rs62541919 | 51657 | 0.659 | chr9 | 16853364 |
| rs3814113 | rs4961501 | 63343 | 0.655 | chr9 | 16841678 |
| rs3814113 | rs7046326 | 67501 | 0.655 | chr9 | 16837520 |
| rs3814113 | rs58691828 | 10718 | 0.651 | chr9 | 16894303 |
| rs3814113 | rs10810655 | 37883 | 0.622 | chr9 | 16867138 |
| rs3814113 | rs10962691 | 84 | 0.609 | chr9 | 16905105 |
| rs3814113 | rs10810670 | 2358 | 0.609 | chr9 | 16902663 |
| rs3814113 | rs10810669 | 2360 | 0.609 | chr9 | 16902661 |
| rs3814113 | rs10810666 | 3355 | 0.609 | chr9 | 16901666 |
| rs3814113 | rs10810665 | 3621 | 0.609 | chr9 | 16901400 |
| rs3814113 | rs62543587 | 4807 | 0.609 | chr9 | 16900214 |
| rs3814113 | rs80039758 | 6638 | 0.609 | chr9 | 16898383 |
| rs3814113 | rs10962662 | 25084 | 0.586 | chr9 | 16879937 |
| rs3814113 | rs10962692 | 853 | 0.581 | chr9 | 16905874 |
| rs3814113 | rs10810668 | 2586 | 0.581 | chr9 | 16902435 |
| rs3814113 | rs117224476 | 7054 | 0.581 | chr9 | 16897967 |
| rs3814113 | rs12344726 | 9693 | 0.577 | chr9 | 16895328 |
| rs3814113 | rs10756835 | 10073 | 0.577 | chr9 | 16894948 |
| rs3814113 | rs7033354 | 10175 | 0.577 | chr9 | 16894846 |
| rs3814113 | rs7033194 | 10316 | 0.577 | chr9 | 16894705 |
| rs3814113 | rs7032175 | 10941 | 0.577 | chr9 | 16894080 |
| rs3814113 | rs62543585 | 8132 | 0.553 | chr9 | 16896889 |
| rs3814113 | rs10962641 | 65417 | 0.548 | chr9 | 16839604 |
| rs3814113 | rs1339547 | 68761 | 0.548 | chr9 | 16836260 |
| rs3814113 | rs7033061 | 10525 | 0.547 | chr9 | 16894496 |
| rs3814113 | rs7032581 | 10666 | 0.547 | chr9 | 16894355 |
| rs3814113 | rs7868157 | 63044 | 0.546 | chr9 | 16841977 |
| rs3814113 | rs77795022 | 7024 | 0.537 | chr9 | 16897997 |
| rs3814113 | rs62543582 | 8927 | 0.537 | chr9 | 16896094 |
| rs3814113 | rs12379183 | 49322 | 0.526 | chr9 | 16855699 |
| rs3814113 | rs7861573 | 52741 | 0.526 | chr9 | 16852280 |
| rs3814113 | rs7033084 | 10380 | 0.516 | chr9 | 16894641 |
| rs3814113 | rs1339548 | 68698 | 0.516 | chr9 | 16836323 |
| rs3814113 | rs4961498 | 72008 | 0.515 | chr9 | 16833013 |
| rs3814113 | rs7868583 | 8662 | 0.513 | chr9 | 16896359 |
| rs3814113 | rs7032420 | 10819 | 0.513 | chr9 | 16894202 |
| rs3814113 | rs72713890 | 8511 | 0.509 | chr9 | 16896510 |
| rs3814113 | rs62543584 | 8714 | 0.509 | chr9 | 16896307 |
| rs3814113 | rs62543583 | 8869 | 0.509 | chr9 | 16896152 |
| rs3814113 | rs62543581 | 9015 | 0.509 | chr9 | 16896006 |
| rs3814113 | rs117376794 | 5546 | 0.5 | chr9 | 16899475 |
| rs3814113 | rs62543578 | 10386 | 0.497 | chr9 | 16894635 |
| rs3814113 | rs7032644 | 10880 | 0.464 | chr9 | 16894141 |
| rs3814113 | rs62543561 | 15736 | 0.456 | chr9 | 16889285 |
| rs3814113 | rs10962666 | 22749 | 0.441 | chr9 | 16882272 |
| rs3814113 | rs10810645 | 69295 | 0.436 | chr9 | 16835726 |
| rs3814113 | rs12551733 | 19443 | 0.424 | chr9 | 16885578 |
| rs3814113 | rs10810661 | 11171 | 0.421 | chr9 | 16893850 |

SNP dataset from 1000 Genome Pilot 1. Population panel CEU. r^2^ threshold ≥ 0.4. In bold SNPs with a correlation ≥ 0.8.

Supplementary Table 2

| **Cell line name** | **Source ^#^** | **Species** | **Biological source** | **Medium^§^** |
| --- | --- | --- | --- | --- |
| SKOV3 | ATCC (HTB-77™) | H. sapiens | Serous ovarian cancer | RPMI, 10% FBS |
| OV90 | ATCC (CRL-11732™) | H. sapiens | Serous ovarian cancer | MEDIUM 199, NaB, 15% FBS |
| FUOV1 | DSMZ | H. sapiens | Serous ovarian cancer | DMEM : HAM'S F12 (1:1), NaP, Gln, 15% FBS |
| COV318 | ECACC | H. sapiens | Serous ovarian cancer | DMEM, NaP, Gln, 10% FBS |
| COV504 | ECACC | H. sapiens | Serous ovarian cancer | DMEM, NaP, Gln, 10% FBS |
| OAW42 | ECACC | H. sapiens | Serous ovarian cancer | DMEM, NaP. Gln, insulin, 10% FBS |
| OV56 | ECACC | H. sapiens | Serous ovarian cancer | DMEM : HAM'S F12 (1:1), NaP, Gln, Hydrocortisone, insulin, 5% FBS |
| OC 316 | ICLC | H. sapiens | Serous ovarian cancer | RPMI, 5% FBS |
| IGROV1 | NCI/DCTD | H. sapiens | Serous ovarian cancer | RPMI, 10% FBS |
| OVCAR4 | NCI/DCTD | H. sapiens | Serous ovarian cancer | RPMI, 10% FBS |
| OVCAR8 | NCI/DCTD | H. sapiens | Serous ovarian cancer | RPMI, 10% FBS |
| KURAMOCHI | HSRRB | H. sapiens | Poorly differentiated ovarian cancer | RPMI, 10% FBS |
| OVSAHO | HSRRB | H. sapiens | Ovarian adenocarcinoma | RPMI, 10% FBS |
| COV362 | ECACC | H. sapiens | Endometrioid ovarian carcinoma | DMEM, NaP, Gln, 10% FBS |
| TOV21G | ATCC (CRL-11730™) | H. sapiens | Clear cell ovarian carcinoma | RPMI, 10% FBS |
| TOV112D | ATCC (CRL-11731™) | H. sapiens | Endometrioid ovarian carcinoma | RPMI, 10% FBS |
| HCT116 | ATCC (CCL-247™) | H. sapiens | Colorectal carcinoma | RPMI, 10% FBS |
| U2OS | Sigma-Aldrich | H. sapiens | Osteosarcoma | DMEM, Gln, 10% FBS |
| 293FT | Thermo Fisher Scientific | H. sapiens | Embryonal kidney | DMEM, Gln, NaP, NEAA, 10% FBS |

**# ATCC** (http://www.atcc.org/); **DSMZ** (http://www.dsmz.de/); **ECACC** (http://www.hpacultures.org.uk/collections/ecacc.jsp); **HSRRB** (http://www.jhsf.or.jp/English/hsrrb.html); **ICLC** (http://www.iclc.it/Listanuova.html); **NCI/DCTD** (http://dtp.nci.nih.gov/branches/btb/tumor-catalog.pdf)

**§ RPMI** (RPMI-1640 AQmedia), **DMEM** (DMEM AQmedia), **HAM’S F12**, **MEDIUM 199,** **NaB** (Sodium Bicarbonate), **NaP** (Sodium Pyruvate), **Gln** (Glutamine), **NEAA** (Non- Essential Amino Acids), **Insulin** (9.5 - 11.5 μg/ml), **Hydrocortisone** (0.5 μg/ml), **Transferrin** (5 μg/ml) and **Sodium Selenite** (5 ng/ml) were all from Sigma-Aldrich (St. Louis. MO. USA). **FBS** (Fetal Bovine Serum) was from Gybco (Carlsbad CA, USA).

Supplementary Table 3

| **Primer name** | **Primer sequence (5’-3’)** |
| --- | --- |
| screen22 5 | TTTATTTTGGACATCATAAACCTTTT |
| screen22 3 | ACGAGGAAAGCCAGAGACCT |
| Guide 6 S | CACCGATTCAGCAGTGTTAGAACTG |
| Guide 6 AS | AAACCAGTTCTAACACTGCTGAATC |
| Guide 9 S | CACCGCTAGAGATGTGACCAAGGAC |
| Guide 9 AS | AAACGTCCTTGGTCACATCTCTAGC |
| AK024561 5 | TTTCCTGTTTTCCTCCATGC |
| AK024561 3 | CCAGTTGGAGGTGAGTTCGT |
| Screen 6F | CCATACCCAAACCTGAAGGA |
| Screen 7R | GCCCGGCCTACTTTATCATC |
| BNC2 exon6^#^ 5 | GGGGATTCTTCTCGATGACA |
| BNC2_exon6^#^ 3 | ACTCTCAGGGTCCCCTTGTT |
| BNC2_exon1-2^#^ 5 | CCGCTAAGAGGAGACCAAGA |
| BNC2_exon1-2^#^ 3 | CACAACATGGGACCTTGAAA |
| BNC2_exon2-2a^#^ 5 | CCCACCTCCACATAGCCTTA |
| BNC2_exon2-2a^#^ 3 | GTTCTGGTTCCGAACTGCAT |
| BNC2_232568a 5 | CTACCCCTGTGGCACTGTTT |
| BNC2_232568a 3 | CACCCCAGCAGCCATATACT |
| BNC2_232568b 5 | ATGTCACTCAGCCCCAGTTC |
| BNC2_232568b 3 | GCCAGCACCTTCATCTCTTC |
| BNC2_232568c 5 | GAGGAGCAGCTGAAAAATGC |
| BNC2_232568c 3 | GGTGGGAGTGAACTCTTGGA |
| BNC2_232568d 5 | CAGAAAATGGCTGCTTTTCC |
| BNC2_232568d 3 | TCGTCTGTTTGAACCACCAA |
| BNC2_232568e 5 | CACGCAGACACAAGACATCC |
| BNC2_232568e 3 | TCGTCTGTTTGAACCACCAA |
| BNC2_Intr2 5 | TGGGAACAGAATTGCCATAA |
| BNC2_Intr2 3 | CCACCCCCAGCACTATTTTA |
| BNC2_Enh1 5 | AGTTGGGGCCCTTAAAAGAA |
| BNC2_Enh1 3 | ATGTGTGGTTGCCTTGTTGA |
| BNC2_Prom2 5 | TCGGTATCCTTTCCCCTTTT |
| BNC2_Prom2 3 | ATGGGCACTTTTGTTTCTGG |
| BNC2_Enh2 5 | CCAGCAGGAGTGATGACTGA |
| BNC2_Enh2 3 | GGTTCCCTCTGTGGCTCATA |
| BNC2_Prom 5 | TGCAAACACCACCCAACTTA |
| BNC2_Prom 3 | GTTCCCAGAGTGCGCTGTAT |
| Intergenic Enh1 5 | AAGGAGCCCATTCAGGTCTT |
| Intergenic Enh1 3 | GTGCAACATGAAACCAATGC |
| Intergenic Enh2 5 | TGGAAAGGGCAGAGCTTAAA |
| Intergenic Enh2 3 | TTTCCCCCACTACTCACAGG |
| Intergenic Enh3 5 | AAAGCCTCTTTTCTGGCACA |
| Intergenic Enh3 3 | AGAGGGCAGCCTCTCCTAAC |
| CNTLN 5 | GTAGAGGAGGCGATGGTGAC |
| CNTLN 3 | TTCCACAACCAAACTGACCA |
| CNTLN Prom 5 | AACAGGGCTGGGGAGATTAG |
| CNTLN Prom 3 | CCCACCTCTTAAAGCCTTCC |
| GAPDH 5 | GAGAGACCCTCACTGCTG |
| GAPDH 3 | GATGGTACATGACAAGGTGC |
| U6 5 | CTCGCTTCGGCAGCACA |
| U6 3 | AACGCTTCACGAATTTGCGT |

^#^ Nomenclature of BNC2 Exons is reported according to (3).

# Supplementary Figure S1.

**
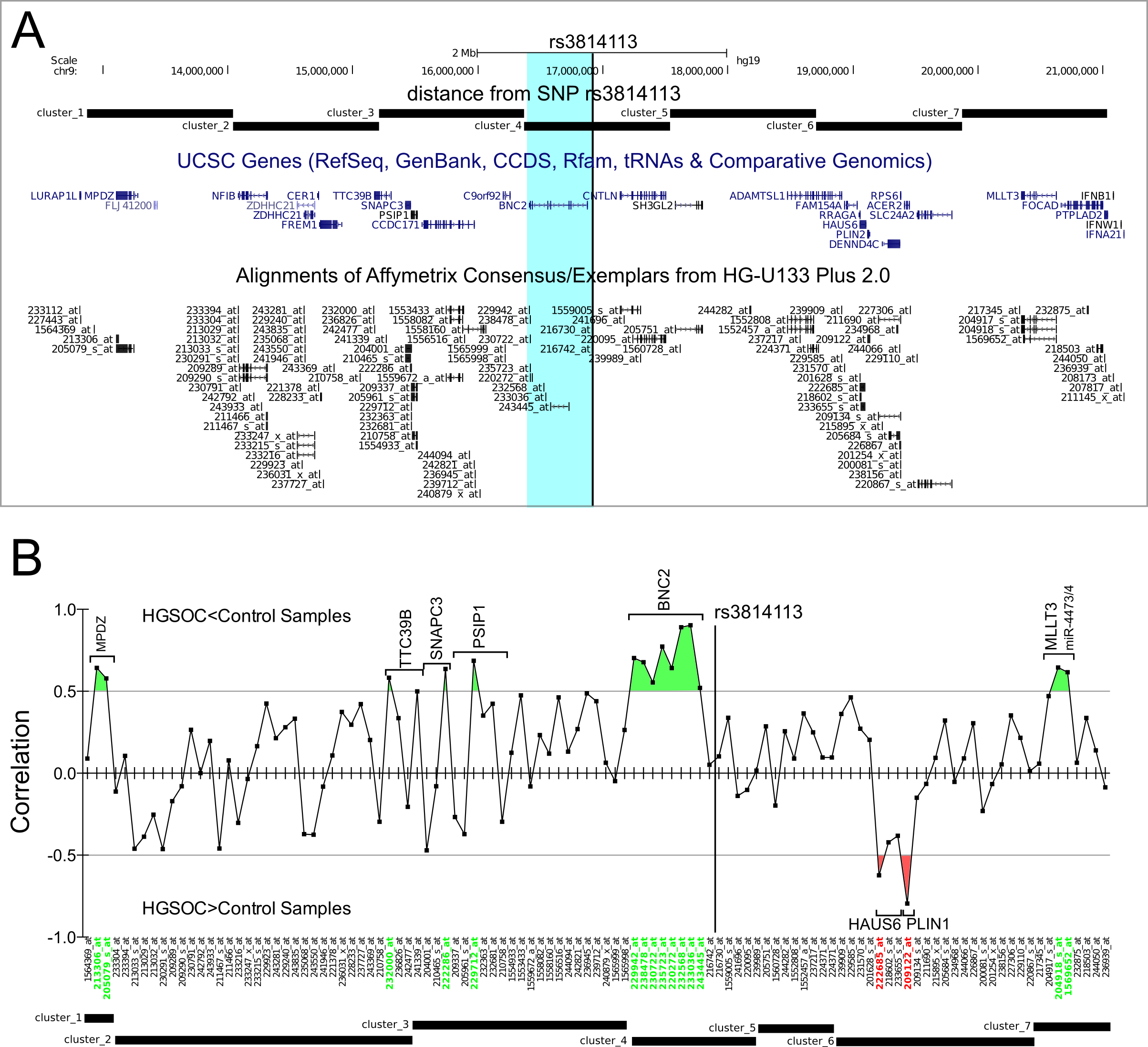
**

**Supplementary Figure S1.** (**A**) Genomic view of rs3814113 (vertical line) ± 4 Mb of human 9p22.2, showing the alignment of annotated human genes (in blue) and of the probes from Affymetrix Human Genome U133 Plus 2.0 Array (in black), according to UCSC genome browser. Cluster_1 to cluster_7 indicate the genetic blocks of approximately 1.2 Mb in which the Affymetrix probes (n= 150, covering 30 genes and associated intergenic regions) have been grouped; in the middle of cluster_4 is located rs3814113; highlighted in light blue the BNC2 genomic locus. (**B**) Correlation of signal intensities of the Affymetrix probes indicated in (A) from GSE10971 dataset (2) with sample type (i.e. HGSOC or Fallopian Tubes Epithelium -FTE). Correlation coefficient greater than 0.0 indicates that probe signal intensity is higher in FTEs; correlation coefficient smaller than 0.0 indicates that probe signal intensity is higher in HGSOC. In green and in red are indicated the probes that show a coefficient greater or smaller than ±0.5, i.e. that have a lower or higher signal intensity in HGSOC cases, respectively. The correlation was evaluated by Pearson test.

# Supplementary Figure S2.

**
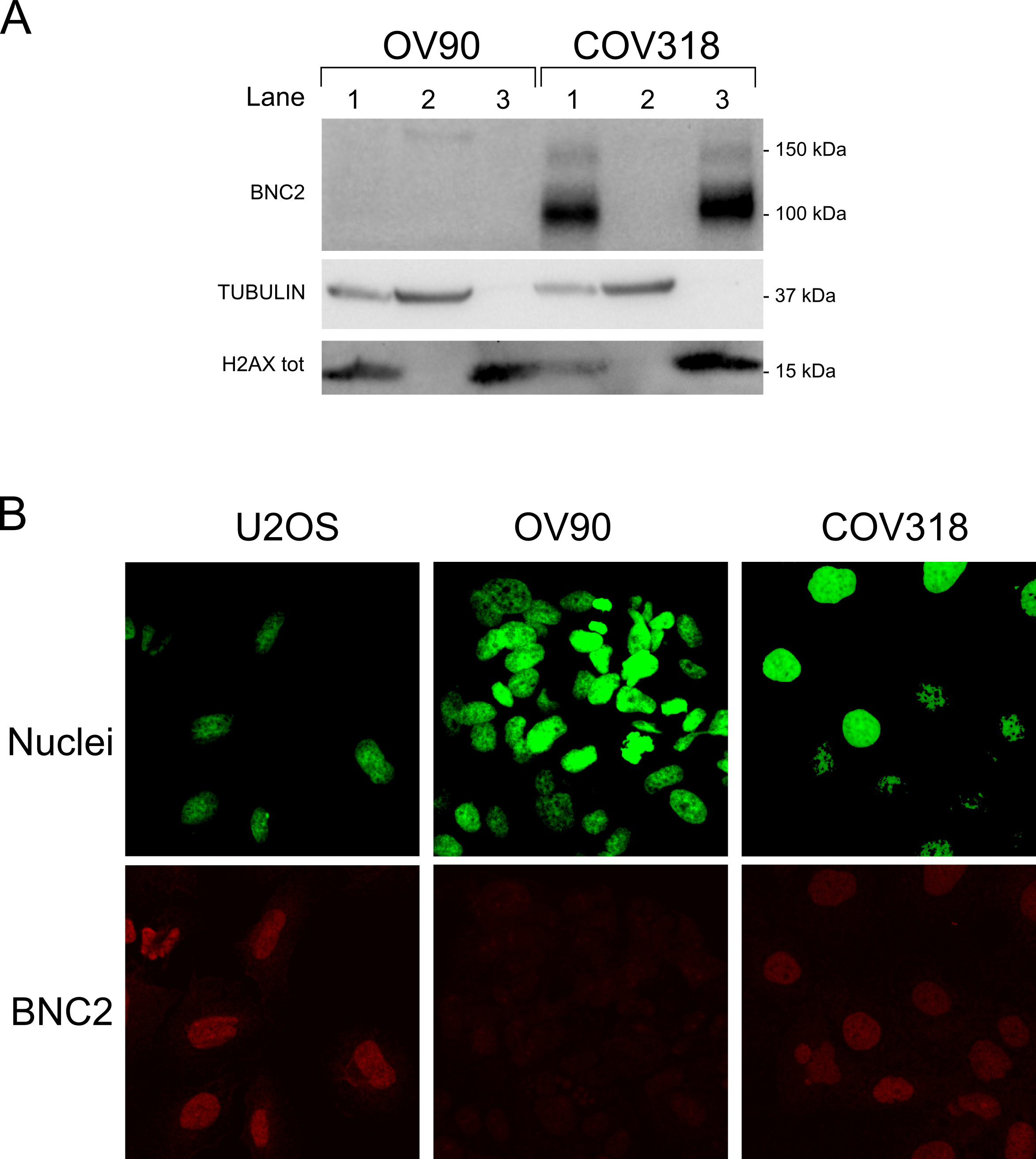
**

**Supplementary Figure S2 (A)** Western blot detection of BNC2 protein levels in SDS lysis buffer total extracts (lane 1), in NP40 soluble extracts (lane 2) and in NP40 insoluble extracts (lane 3) from OV90 and COV318. Tubulin and H2AX total expression were used to confirm the separation between cytosolic/soluble and chromatin/insoluble fractions, respectively. **(B)** BNC2 immunostaining of U2OS, OV90 and COV318. Immunostaining was performed following Protein Atlas standard protocol. Antibodies used were: anti-BNC2 (cat no. HPA018525, Sigma-Aldrich) and Alexa Fluor 568-conjugated secondary anti-rabbit (cat no. A-11011, Molecular Probes, Eugene, OR, USA). Nuclei were counterstained with TO-PRO-3 Iodide (Molecular Probes).

# Supplementary Figure S3.


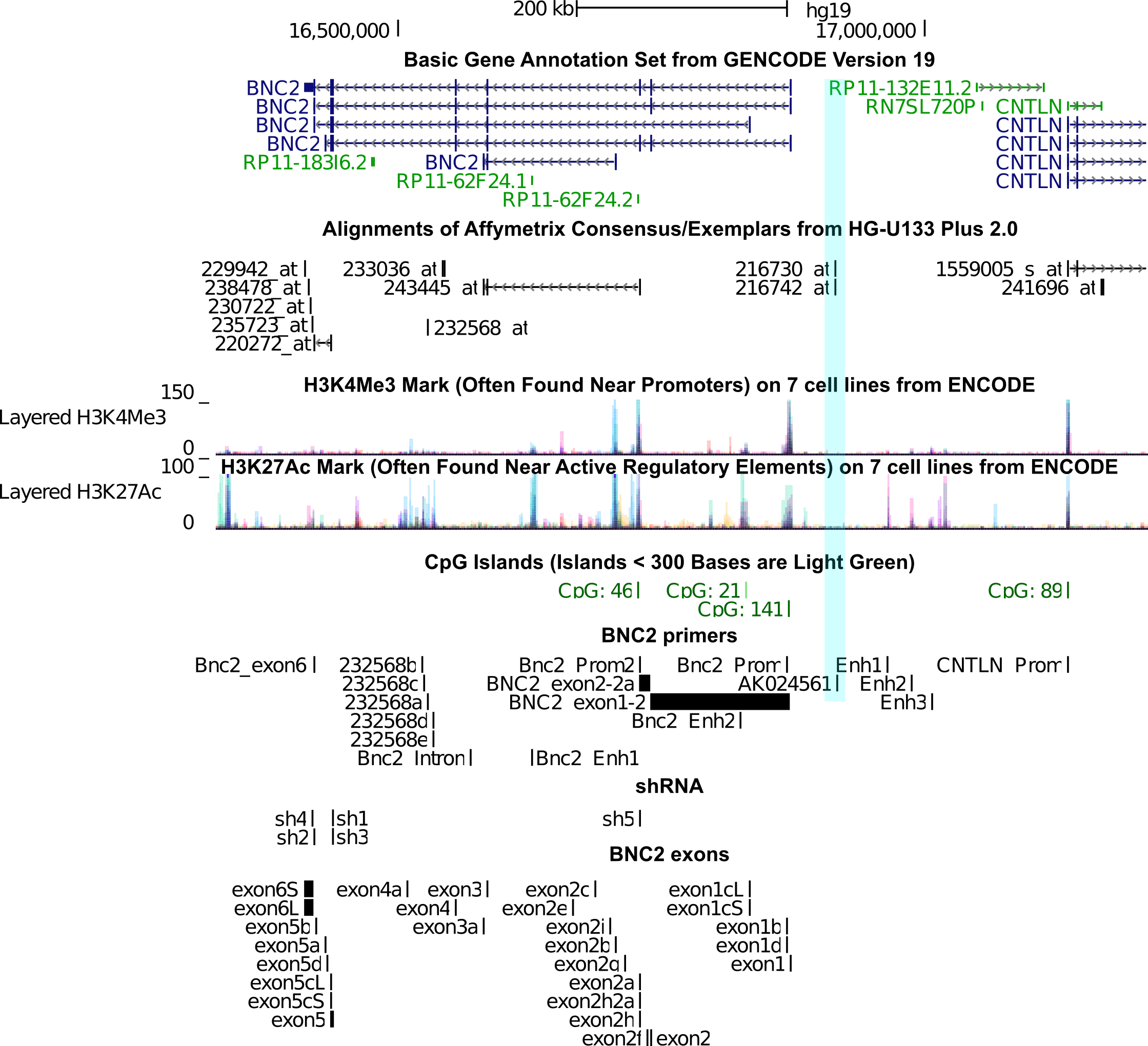


**Supplementary Figure S3**. Genomic view of human 9p22.2 comprising: BNC2 locus, rs3814113 (light blu vertical line) and CNTLN 5’ end, according to UCSC. From top to bottom are shown: annotated genes from GENCODE v19; Affymetrix probes in U133Plus2 chip; histone marks (H3K4Me3 and H3K27Ac) from ENCODE; CpG islands; alignment of the amplicons obtained with the primer pairs indicated in Supplemental table 3; alignment of the short-hairpin sequences used to silence BNC2 and alignment of BNC2 exons according to (3).

# Supplementary Figure S4.


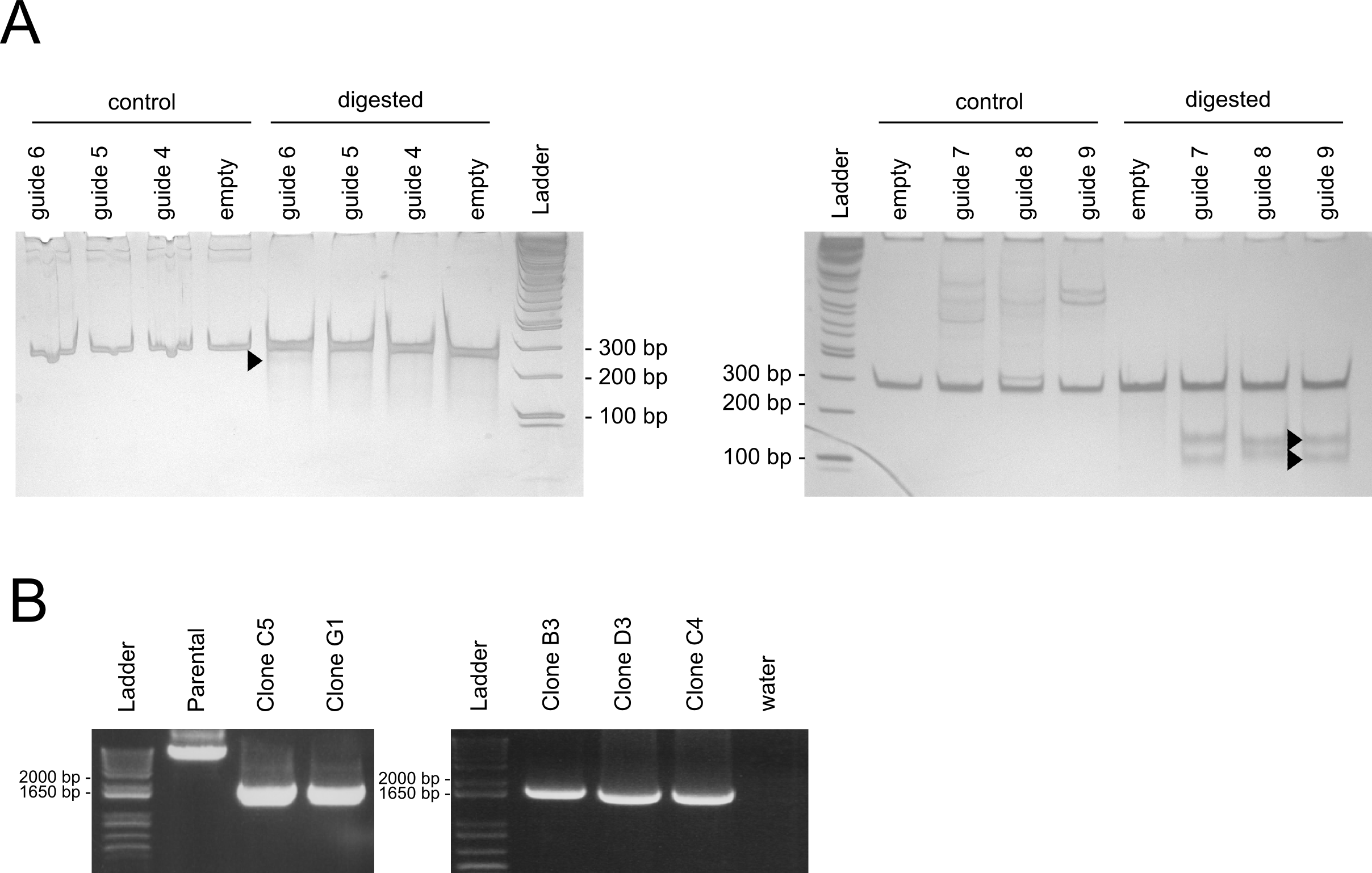


**Supplementary Figure S4.** **(A)** Silver staining of polyacrylamide gels showing surveyor assay that was used to confirm that guide RNA 6 and 9 were effective in generating NHEJ errors in the pool of guide RNA Cas9 cotransfected cells. Black triangles indicate extra bands generated by nuclease cleavage and confirm the existence of mismatching. **(B)** Agarose gels of PCR products using Screen 6F + Screen 7R primer pair (Figure 3A and Supplementary table 3) showing the deletion of the 5 kb region surrounding rs3814113. Parental cells represent cells that were not transfected with guide RNAs and Cas9 plasmids. C5, G1, B3, D3 and C4 are the five deleted clones used in Figure 3D.

# Supplementary Figure S5.


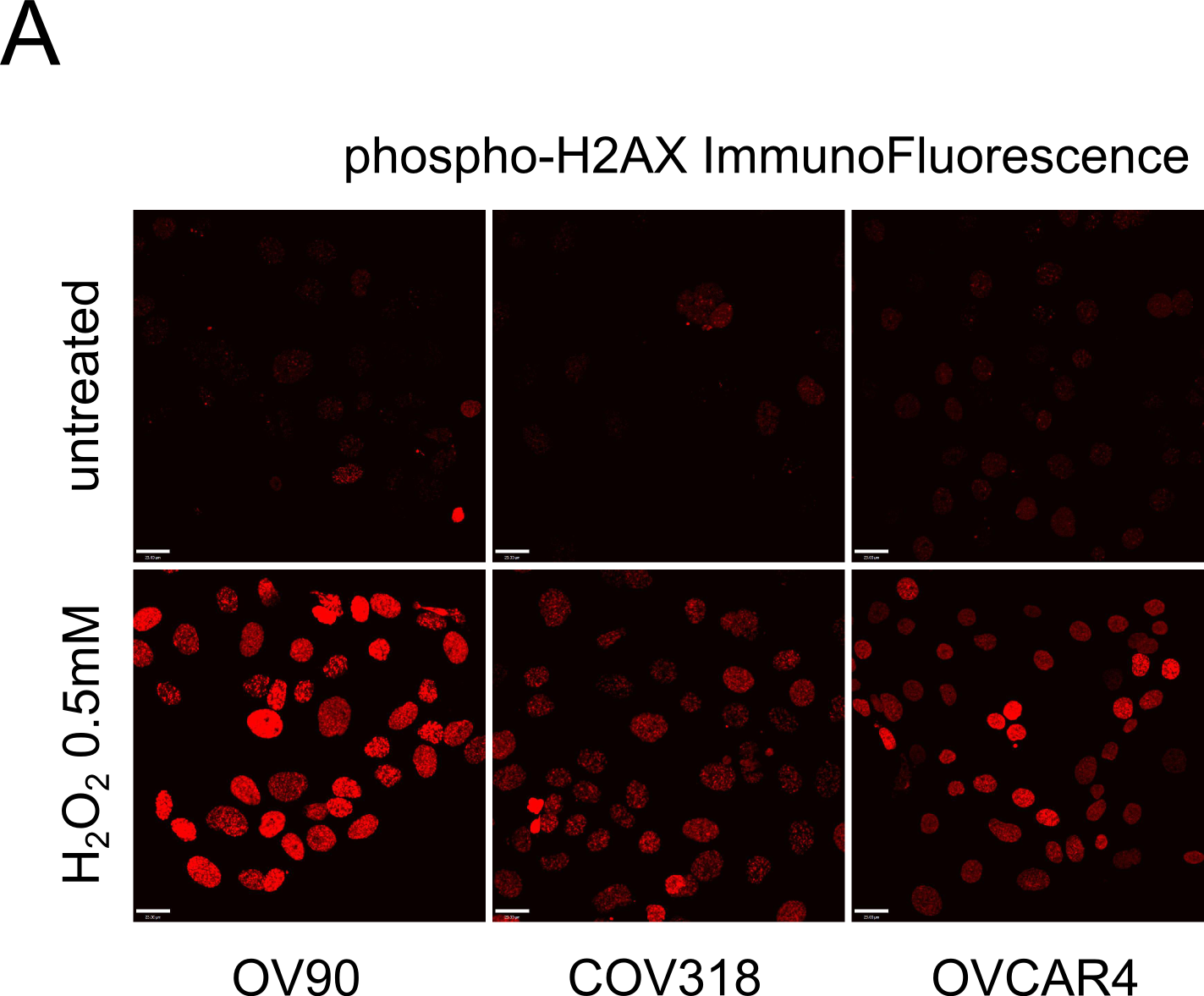


**Supplementary Figure S5.** Phospho-H2AX immunostaining of OV90, COV318 and OVCAR4 cells untreated or treated with 0.5 mM H_2_O_2_, 5 h after treatment. Immunostaining was performed following Protein Atlas standard protocol. Antibodies used were: Phospho-Histone H2AX (Ser139) (cat no. #9718 Cell Signaling) and Alexa Fluor 568-conjugated secondary anti-rabbit (cat no. A-11011, Molecular Probes, Eugene, OR, USA). Nuclei were counterstained with TO-PRO-3 Iodide (Molecular Probes).

# Supplementary Figure S6.

**
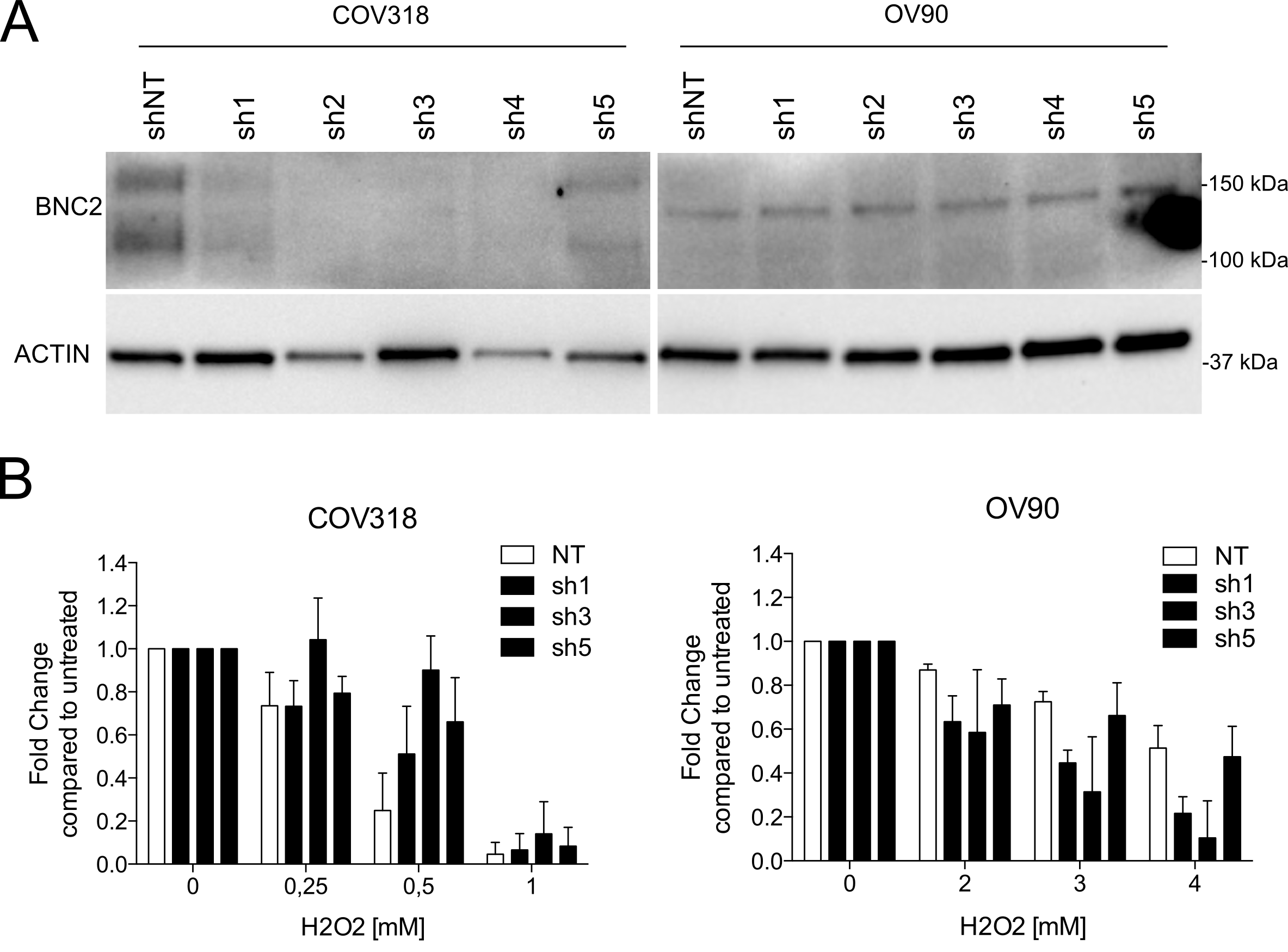
**

**Supplementary Figure S6**. **(A)** Western blot detection of BNC2 protein levels in NP40 insoluble extracts in COV318 and OV90 expressing 5 different short hairpins (sh) against BNC2. shNT= sh non-target, negative control. Actin expression was used as loading control **(B)** MTT viability, expressed as fold change compared to untreated cells (= 1), of COV318 and OV90 24 h following H_2_O_2_ treatment. Bars in graph represent average ±SD of 3 independent biological replicates.

# Supplementary References

1) Roby, K. F., Taylor, C. C., Sweetwood, J. P., Cheng, Y., Pace, J. L., Tawfik, O., Persons, D. L., Smith, P. G., and Terranova, P. F. (2000). Development of a syngeneic mouse model for events related to ovarian cancer. Carcinogenesis 21, 585-591.

2) Tone, A. A., Begley, H., Sharma, M., Murphy, J., Rosen, B., Brown, T. J., and Shaw, P. A. (2008). Gene expression profiles of luteal phase fallopian tube epithelium from BRCA mutation carriers resemble high-grade serous carcinoma. Clinical cancer research: an official journal of the American Association for Cancer Research 14, 4067-4078.

3) Vanhoutteghem, A., and Djian, P. (2007). The human basonuclin 2 gene has the potential to generate nearly 90,000 mRNA isoforms encoding over 2000 different proteins. Genomics 89, 44-58.

**Sanger Sequences of CRISPR clones**

**Seq_ref_guide 6 (UPPERCASE: predicted guide target site)**

caggtactgacagcatagtttctttcttatcatcttcttggtgttccttcagcctcacaagctacagaagtggggttgaggctcttatcagacaagctgtctccatgcgctagtcttaaaaaacatagtagataggaataaagtatagaaacaaagaatattcagcagtgttaga**AC**tgtggctcagaaagaatacaaacttgggttgtaaatctgtgcaaaa

**Seq_guide 6F_cloneB1_WT1 (indel: deletion of 3nt)**

CAGGTAGTGACAGCATAGTTTCTTTCTTATCATCTTCTTGGTGTTCCTTCAGTCTCACAAGCTACAGAAGTGGGGTTGAGGCTCTTATCAGACAAGCTGTCTCCATGCGCTAGTCTTAAAAAACATAGTAGATAGGAATAAAGTATAGAAACAAAGAATATTCAGCAGTGTTACTGTGGCTCAGAAAGAATACAAACTTGGGTTGTAAATCTGTGCAAAA

**Seq_guide 6F_cloneC1_WT2 (indel: insertion of 1nt)**

CTGGACTGGAAGCATAGTTTCTTTCTTATCATCTTCTTGGTGTTCCTTCAGCCCCACAAGCTACAGAAGTGGGGTTGAGGCTCTTATCAGACAAGCTGTCTCCATGCGCTAGTCTTAAAAAACATAGTAGATAGGAATAAAGTATAGAAACAAAGAATATTCAGCAGTGTTAGAAACTGTGGCTCAGAAAGAATACAAACTTGGGTTGTAAATCTGTGCAAAA

**Seq_guide 6F_cloneD1_WT3 (no indels)**

TAGGACTGGACAGCATAGTTTCTTTCTTATCATCTTCTTGGTGTTCCTTCAGCCTCACAAGCTACAGAAGTGGGGTTGAGGCTCTTATCAGACAAGCTGTCTCCATGCGCTAGTCTTAAAAAACATAGTAGATAGGAATAAAGTATAGAAACAAAGAATATTCAGCAGTGTTAGAACTGTGGCTCAGAAAGAATACAAACTTGGGTTGTAAATCTGTGCAAAA

**Seq_guide 6F_cloneE1_WT4 (no indels)**

CAGGTACTGACAGCATAGTTTCTTTCTTATCATCTTCTTGGTGTTCCTTCAGCCTCACAAGCTACAGAAGTGGGGTTGAGGCTCTTATCAGACAAGCTGTCTCCATGCGCTAGTCTTAAAAAACATAGTAGATAGGAATAAAGTATAGAAACAAAGAATATTCAGCAGTGTTAGAACTGTGGCTCAGAAAGAATACAAACTTGGGTTGTAAATCTGTGCAAAA

**Seq_ref_guide 9 (UPPERCASE: predicted guide target site)**

tccctttcctcatgtgaatgccttactcaaaaccttcaatgaattctcattgcttacaaaacttaaatccacctcttgattttgcattaaaaactactcacaatccagtCCttggtcacatctctagtagcccctcacttttccttgggaggaatttgttgcttccaccaatcaagtctcttattgctcattattcgacaaactttgatgagtcctttggttggata

**Seq_guide 9R_cloneB1_WT1 (indel: deletion of 11nt)**

TGTCACCGAAGGACTCATCAAGTTTGTCGAATAATGAGCAATAAGAGACTTGATTGGTGGAAGCAACAAATTCCTCCCAAGGAAAAGTGAGGGGCTACTAGAGATGTGGATTGTGAGTAGTTTTTAATGCAAAATCAAGAGGTGGATTTAAGTTTTGTAAGCAATGAGAATTCATTGAAGGTTTTGAGTAAGGCATTCACATGAGGAAAGGG

**Seq_guide 9R_cloneC1_WT2 (no indels)**

TAGCAACCTAAGGACTCATCAAGTTTGTCGAATAATGAGCAATAAGAGACTTGATTGGTGGAAGCAACAAATTCCTCCCAAGGAAAAGTGAGGGGCTACTAGAGATGTGACCAAGGACTGGATTGTGAGTAGTTTTTAATGCAAAATCAAGAGGTGGATTTAAGTTTTGTAAGCAATGAGAATTCATTGAAGGTTTTGAGTAAGGCATTCACATGAGGAAAGGGA

**Seq_guide 9R_cloneD1_WT3 (no indels)**

TATCATCTAAGGACTCATCAAGTTTGTCGAATAATGAGCAATAAGAGACTTGATTGGTGGAAGCAACAAATTCCTCCCAAGGAAAAGTGAGGGGCTACTAGAGATGTGACCAAGGACTGGATTGTGAGTAGTTTTTAATGCAAAATCAAGAGGTGGATTTAAGTTTTGTAAGCAATGAGAATTCATTGAAGGTTTTGAGTAAGGCATTCACTGAAGGAAAGGGA

**Seq_guide 9R_cloneE1_WT4 (no indels)**

TATCCAACCAAAGGACTCATCAAAGTTTGTCGAATAATGAGCAATAAGAGACTTGATTGGTGGAAGCAACAAATTCCTCCCAAGGAAAAGTGAGGGGCTACTAGAGATGTGACCAAGGACTGGATTGTGAGTAGTTTTTAATGCAAAATCAAGAGGTGGATTTAAGTTTTGTAAGCAATGAGAATTCATTGAAGGTTTTGAGTAAGGCATTCACATGAGGAAAGGGA

**Seq_screen22_3_cloneC5_DEL**

CGATGCTCAGACTAAAGAATTAAGTTTGATCTAAACCTGAGATGCAAGTGGGGAAAGCAGGACGGGCAGTGGGGTAAAAACAAAGTCACAAACAGAAATGTGGACAGCGTGTTTGTGAATGATGCCCTCGGAATGCACCACCCAAAGGACTCAATCAAAGTTTGTCGAATAATGAGCAATAAGAGACTTGATTGGTGGAAGCAACAAATTCCTCCCAAGGAAAAGTGAGGGGCTACTAGAGATGTGACCAAGCTGCTGAATATTCTTTGTTTCTATACTTTATTCCTATCTACTATGTTTTTTAAGACTAGCGCATGGAGACAGCTTGTCTGATAAGAGCCTCAACCCCACTTCTGTAGCTTGTGAGACTGAAGGAACACCAAGAAGATGATAAGAAAGAAACTATGCTGTCATGTTTCCTTTCATGACAAAAGGATGCTGTGCTTATCCCATTGAAATAAAAGCAGGAATTAAACAAATACATGGGTTATAATATTTTTCTCTAATAAGAAATATGGAAAATATAAAAGTAGACTTTGAACTCTTAGCATGTTCTATAAAGACATATTACTTTAAAGTCAGAATGAGTCAGCGAAAACGAGGTTGTTCTTAGCTTTTCCTGAAGCTCTGTTTTCTCAAGCATACTCCCAAATATTAGATCCTGAAAAAAAAAGGTTATGATGTCCAAAATAAACCAGCTGGAGCAGAGTAGCACTAATGTACTTTTTATTCATAACTACTTTTCATTCTTACTCTCAGCATGGTAAACCTTAACTGTCCCCAACCCCCCA

**Seq_ screen22_3_cloneG1_DEL**

CTGATGCTCAGACTAAAGATTAAGTTTGATCTAACCTGAGATGCAGTGGGGAAAGCAGGACGGGCAGTGGGGTAAAAACAAAGTCACAAACAGAAATGTGGACAGCGTGTTTGTGAATGATGCCCTCGGAATGCACCACCCAAAGGACTCAATCAAAGTTTGTCGAATAATGAGCAATAAGAGACTTGATTGGTGGAAGCAACAAATTCCTCCCAAGGAAAAGTGAGGGGCTACTAGAGATGTGACCAAGTTCTAACACTGCTGAATATTCTTTGTTTCTATACTTTATTCCTATCTACTATGTTTTTTAAGACTAGCGCATGGAGACAGCTTGTCTGATAAGAGCCTCAACCCCACTTCTGTAGCTTGTGGGGCTGAGGGAACACCAAGAAGATGATAAGAAAGAAACTATGCTGTCATGTTTCCTTTCATGACAAAAGGATGCTGTGCTTATCCCATTGAAATAAAAGCAGGAATTAAACAAATACATGGGTTATAATATTTTTCTCTAATAAGAAATATGGAAAATATAAAAGTAGACTTTGAACTCTTAGCATGTTCTATAAAGACATATTACTTTAAAGTCAGAATGAGTCAGCCAAAACGAGGTTGTTCTTAGCTTTTCCTGAAGCTCTGTTTTCTCAGCATACTCCAAATAATAAGATCCTGGAAAAAAAAATGTTTATGATGTCCAGATAACTAGGCTGAGCAAGCAGCACTTCATGTATCTTGATCGATACTACTTGCCTCGTACTTTCTCAACATGTAAACCTAGAGCTTGGCGAG

**Seq_ screen22_3_cloneB3_DEL**

CTGAGGCTCAGACTAAAGAATTAAGTTTGATCTAACCTGAGATGCAGTGGGGAAAGCAGGACGGGCAGTGGGGTAAAAACAAAGTCACAAACAGAAATGTGGACAGCGTGTTTGTGAATGATGCCCTCGGAATGCACCACCCAAAGGACTCAATCAAAGTTTGTCGAATAATGAGCAATAAGAGACTTGATTGGTGGAAGCAACAAATTCCTCCCAAGGAAAAGTGAGGGGCTACTAGAGATGTGACCAACTGCTGAATATTCTTTGTTTCTATACTTTATTCCTATCTACTATGTTTTTTAAGACTAGCGCATGGAGACAGCTTGTCTGATAAGAGCCTCAACCCCACTTCTGTAGCTTGTGGGGCTGAAGGAACACCGAGAAGATGATAAGAAAGAAACTATGCTGTCATGTTTCCTTTCATGACAAAAGGATGCTGTGCTTATCCCATTGAAATAAAAGCAGGAATTAAACAAATACATGGGTTATAATATTTTTCTCTAATAAGAAATATGGAAAATATAAAAGTAGACTTTGAACTCTTAGCATGTTCTATAAAGACATATTACTTTAAAGTCAGAATGAGTCAGCCAAAACGAGGTTGTTCTTAGCTTTTCCTGAAGCTCTGTTTCTCAGCATACTCCAATATAGATCATGAAAAAAAATGATCATGATGTCGATAATAATCAGCTGGAGGTCAAGTAGCACTGACTGATCTTAATCATTACTACGTTACTCTACGTTCTAGCATTGGTAAACTAACTGGCCTAACCGCCCAAG

**Seq_ screen22_3_cloneD3_DEL**

TTAAGTTTCCCGGCGAGCTCGACTAAGAATTAGTTTGATCTAAACCTGAGATGCAAGTGGGGAAAGCAGGACGGGCAGTGGGGTAAAAACAAAGTCACAAACAGAAATGTGGACAGCGTGTTTGTGAATGATGCCCTCGGAATGCACCACCCAAAGGACTCAATCAAAGTTTGTCGAATAATGAGCAATAAGAGACTTGATTGGTGGAAGCAACAAATTCCTCCCAAGGAAAAGTGAGGGGCTACTAGAGATGTGACCAAGTTCTAACACTGCTGAATATTCTTTGTTTCTATACTTTATTCCTATCTACTATGTTTTTTAAGACTAGCGCATGGAGACAGCTTGTCTGATAAGAGCCTCAACCCCACTTCTGTAGCTTGTGAGACTGAAGGAACACCAAGAAGATGATAAGAAAGAAACTATGCTGTCATGTTTCCTTTCATGACAAAAGGATGCTGTGCTTATCCCATTGAAATAAAAGCAGGAATTAAACAAATAGGGGGGGGTTATAATATTTTTCTCTAATAAGAAATATGGAAAATATAAAAGTAGACTTTGAACTCTTAGCATGTTCTATAAAGACATATTACTTTAAAGTCAGAATGAGTCAGCCAAAACGAGGTTGTTCTTAGCTTTTCCTGAAGCTCTGTTTCTCAAGCATACTCCCAAATATAGATCCTGAAAAAAAAAGGTTTATGATGTCCAAAATAAACAGCTGGGAGGCCAAGAGTAGCCACTCATGTATTTTTGATCCATTAACTACGTTTTCCTCGTACTTTCTTCGAGCAAGTGTTAC

**Seq_ screen22_3_cloneC4_DEL**

TTAGTTGGTCGGCGAGCTCAGACTAAGAATTAAGTTTGATCTAAACCTGAGATGCAAGTGGGGAAAGCAGGACGGGCAGTGGGGTAAAAACAAAGTCACAAACAGAAATGTGGACAGCGTGTTTGTGAATGATGCCCTCGGAATGCACCACCCAAAGGACTCAATCAAAGTTTGTCGAATAATGAGCAATAAGAGACTTGATTGGTGGAAGCAACAAATTCCTCCCAAGGAAAAGTGAGGGGCTACTAGAGATGTGACCAAGTTCTAACACTGCTGAATATTCTTTGTTTCTATACTTTATTCCTATCTACTATGTTTTTTAAGACTAGCGCATGGAGACAGCTTGTCTGATAAGAGCCTCAACCCCACGTCTGTAGCTTGTGAGACTGAAGGAACACCAAGAAGATGATAAGAAAGAAACTATGCTGTCATGTTTCCTTTCATGACAAAAGGATGCTGTGCTTATCCCATTGAAATAAAAGCAGGAATTAAACAAATACATGGGTTATAATATTTTTCTCTAATAAGAAATATGGAAAATATAAAAGTAGACTTTGAACTCTTAGCATGTTCTATAAAGACATATTACTTTAAAGTCAGAATGAGTCAGCCAAAACGAGGTTGTTCTTAGCTTTTCCTGAAGCTCTGTTTTCTCAAGCATACTCCCAAATATTAGATCCTGAAAAAAAAGGTTTATGATGTCCCAAAATAAACCAGCTGGAGGCCAAGAGTAGCACTTCATGTATTTTTATCCATACCTACGTTTTCCCTCCTACTTCCTCAGAGCAGTGTT
